# Parvalbumin expression in oligodendrocyte-like CG4 cells causes a reduction in mitochondrial volume, attenuation in reactive oxygen species production and a decrease in cell processes’ length and branching

**DOI:** 10.1038/s41598-019-47112-9

**Published:** 2019-07-22

**Authors:** Lucia Lichvarova, Walter Blum, Beat Schwaller, Viktoria Szabolcsi

**Affiliations:** Anatomy, Section of Medicine, University of Fribourg, Route Albert-Gockel 1, CH-1700 Fribourg, Switzerland

**Keywords:** Cellular neuroscience, Mitochondria

## Abstract

Forebrain glial cells - ependymal cells and astrocytes -acquire upon injury- a “reactive” phenotype associated with parvalbumin (PV) upregulation. Since free radicals, e.g. reactive oxygen species (ROS) play a role in the pathogenesis of multiple sclerosis, and that PV-upregulation in glial cells is inversely correlated with the level of oxidative stress, we hypothesized that PV-upregulation might also protect oligodendrocytes by decreasing ROS production. Lentiviral transduction techniques allowed for PV overexpression in CG4 oligodendrocyte progenitor cells (OPCs). Depending on the growth medium CG4 cells can be maintained in an OPC-like state, or induced to differentiate into an oligodendrocyte (OLG)-like phenotype. While increased levels of PV had no effect on cell proliferation and invasiveness *in vitro*, PV decreased the mitochondria volume in CG4 cell bodies, as well as the mitochondrial density in CG4 processes in both OPC-like and OLG-like states. In line with the PV-induced global decrease in mitochondrial volume, elevated PV levels reduced transcript levels of mitochondrial transcription factors involved in mitochondria biogenesis. In differentiated PV-overexpressing CG4 cells with a decreased mitochondrial volume, UV-induced ROS production was lower than in control CG4 cells hinting towards a possible role of PV in counteracting oxidative stress. Unexpectedly, PV also decreased the length of processes in undifferentiated CG4 cells and moreover diminished branching of differentiated CG4 cell processes, strongly correlated with the decreased density of mitochondria in CG4 cell processes. Thus besides conferring a protective role against oxidative stress, PV in a cell autonomous fashion additionally affects process’ growth and branching in CG4 cells.

## Introduction

Oligodendrocytes (OLGs) are glial cells with diverse functions in the vertebrate central nervous system (CNS), one crucial being the myelination of long projection axons^1^. In addition to this fundamental task, OLGs play an essential role in neural development and synaptic plasticity^2^. In pathological conditions, OLGs are implicated in demyelinating diseases^3^. The myelinating OLGs derive from migratory oligodendrocyte precursor cells (OPCs). OPCs go through a step-by-step phenotypic and genotypic differentiation program from originally bipolar progenitor cells to the ones able to build the myelin sheath surrounding the axons^4,5^. With respect to morphological development they closely resemble neurons that often start out as bipolar cells, before developing the highly branched neuropil (dendrites and axons) characteristic for multipolar neurons^6^.

Multiple sclerosis (MS) is characterized by the formation of multiple lesions in the CNS in which myelin is destroyed and myelin-producing OLGs perish^7^. Inflammatory mediators produced by infiltrating leukocytes lead to the production of reactive oxygen species (ROS), and play a crucial role in the pathogenesis of MS, since excessive release of free radicals by macrophages, microglia and astrocytes contributes to axonal degeneration and myelin damage^8,9^.

Oligodendrocytes are especially vulnerable to oxidative stress, hypoxia, excitatory amino acids, activation of apoptotic pathways, and deprivation of neurotrophic factors^10^. They are more sensitive to hypoxic conditions than astrocytes, microglia or endothelial cells, and they are as sensitive to ischemia as neurons^11^. Glutathione synthesis and redox regulation play critical roles in the myelination process in both rodents and humans^12,13^. Oligodendrocyte dysfunction is the direct cause of demyelinating lesions in the CNS, and the impaired energy metabolism mediated by damaging of mitochondrial DNA, mitochondrial membrane and mitochondrial respiratory chain complexes might be involved in this process^14^.

Parvalbumin (PV) is a calcium-binding protein of the EF-hand family that hallmarks subpopulations of GABA-ergic interneurons in the human, as well as in the murine brain^15^. Alterations of inhibitory interneurons include an impairment (weakening) of the GABA-ergic phenotype (e.g. decrease of PV and GAD67 expression), as well as a reduction in the number of PV-positive (PV+) interneurons^16^ and a decrease in PV protein levels in neurons under pathological conditions such as schizophrenia^17^ and autism spectrum disorder (ASD)^18^. In normal conditions, there is no significant PV expression, neither in glial cells nor in progenitor cells. However, following brain injury we observed upregulation of PV in ependymal cells and reactive astrocytes. Ependymal cells react with prompt and persistent PV-upregulation to invasive injury inflicted onto the lateral ventricle wall^19^ and in aging-related lateral ventricle wall stenosis^20^. We demonstrated that injury-induced PV overexpression in ependymal cells was dependent on NF-κB signaling and was modulated by altering the level of oxidative stress, which implicates inflammation induced by injury in this process. We postulated that injury-induced PV-upregulation in other glial cells might also protect against oxidative stress and promote regeneration, and that glial PV-upregulation might also occur upon diverse inflammatory processes that involve NF-κB signaling and responses to oxidative stress, such as those observed during the pathogenesis of MS.

In the present study, we used lentiviral-mediated upregulation of PV in rat CG4 cells to study its effect on cell proliferation, migration and cell morphology in non-differentiated and differentiated oligodendroglial cells. The CG4 cell line is a well-characterized line resembling OPCs *in vitro* in non-differentiating medium, with the potential of differentiation into mature OLG-like cells^21^. Given the primary function of PV in Ca^2+^ buffering and previous findings demonstrating an inverse reciprocal relation between PV expression levels and mitochondrial volume in muscle cells^22^, Purkinje cells^23^ and renal epithelial cells^24,25^, we were particularly interested in putative changes in mitochondrial volume and mitochondria-associated changes in cell morphology with a special focus on ROS production and resistance to hypoxia. Assuming a PV upregulation-mediated decrease in mitochondrial volume, we investigated the extent of mitochondria-mediated oxidative stress in undifferentiated CG4 cells resembling OPCs and differentiated CG4 cells resembling OLGs.

## Materials and Methods

### Cell culture

Control (C-) CG4 and B104 cells were a kind gift of Catherine Lubetzki (ICM - Brain and Spine Institute, Paris, France). Cells were grown in tissue culture dishes previously coated with 20 µg/ml poly-L-ornithine (P3655, Sigma-Aldrich, Buchs, Switzerland). Cells were cultivated in proliferation medium composed of 70% N1 medium with biotin [DMEM (D5796, Sigma-Aldrich, Buchs, Switzerland) with N1 supplement (N6530, Sigma-Aldrich) containing 5 µg/ml insulin, 5 µg/ml transferrin, 5 ng/ml sodium selenite, 16 µg/ml putrescine, 7.3 ng/ml progesterone and 10 ng/ml biotin (Acros Organics)] and 30% B104-conditioned N1 medium (DMEM + N1 supplement). In order to induce the differentiation of progenitor cells *in vitro*, cell cultures were switched to differentiation medium consisting of N1 medium (DMEM + N1 supplement) with 10 ng/ml biotin. B104 cells were grown in medium consisting of DMEM + 10% fetal bovine serum until they reached approximately 80% confluence. Afterwards serum-containing medium was removed, cells were rinsed with Phosphate-buffered saline (PBS) and N1 medium was added to the flasks. B104-conditioned N1 medium was collected after 4–5 days, sterile-filtered and stored at −20 °C until use.

### Vector construction and lentivirus production

pLVTHM (Addgene plasmid #12247) was a kind gift of Prof. D. Trono (EPFL, Switzerland) and pLV-PVALB as well as pLKO.1 shRNA PVALB #22 were described previously^24^. Lentivirus for pLVTHM, pLV-PVALB and pLKO.1 shRNA PVALB #22 were produced as described before^26^.

### Transduction and engineering of Pvalb-expressing CG4 cells and CG4 shRNA for Pvalb

CG4 rat oligodendrocyte precursor cells were seeded in 24-well plates (20,000 cells/well) and transduced with lentivirus (MOI between 5 and 10). pLKO.1-shRNA PVALB cells were selected with 2 µg/mL of puromycin (Sigma-Aldrich, Switzerland) for 1 week. pLVTHM cells were checked for enhanced green fluorescent protein (eGFP) expression under the fluorescent microscope (more than 95% of cells were eGFP-positive).

### RNA isolation and semi-quantitative or quantitative reverse transcription polymerase chain reaction (RT-PCR and RT-qPCR)

**Figure 1:**
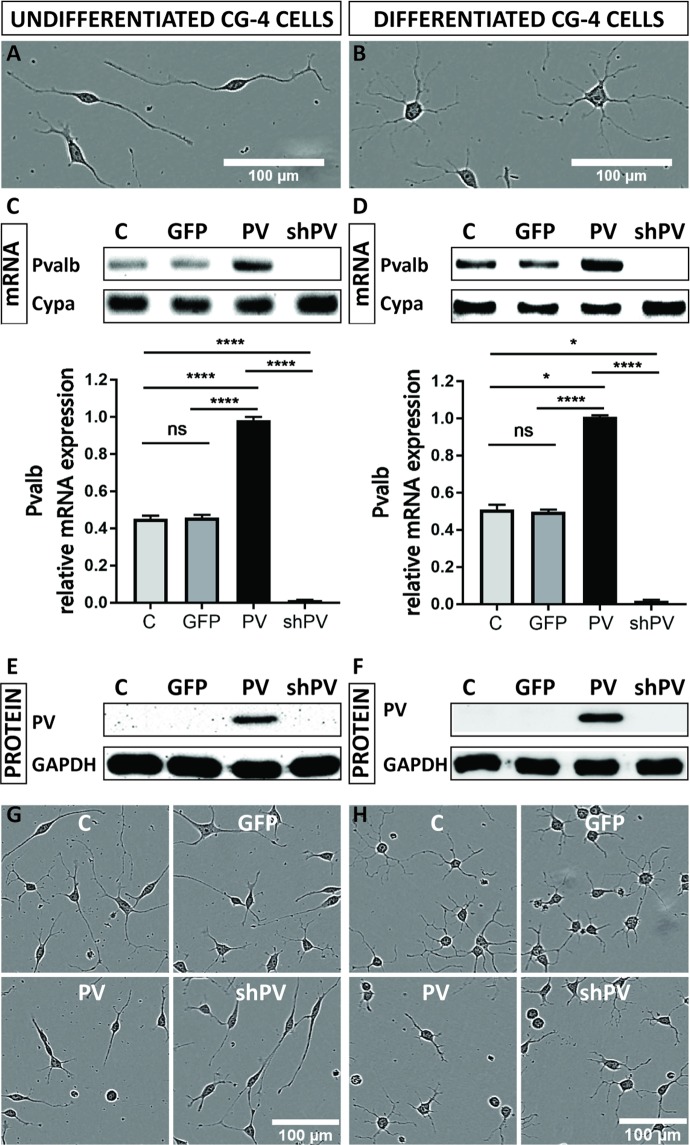
CG4 cells display an OPC-like morphology in the undifferentiated state (**A**) and oligodendrocyte-like phenotype in the differentiated state (**B**). PV (*Pvalb*) transcript levels were determined by semi-quantitative PCR after 30 cycles (upper) and by RT-qPCR (lower), where *Cypa* was used as reference gene (**C**,**D**). PV expression levels were determined by Western blot analyses, where GAPDH signals were used as loading control (**E**,**F**). The morphological features of LV-infected CG4 cells (GFP, PV, sh*PV*) appeared similar to the control (C) in both undifferentiated OPC-like and differentiated OLG-like states (**G**,**H**).

To investigate *Pvalb* mRNA expression levels in a semi-quantitative way, total RNA was extracted from 70–90% confluent cell cultures (75 cm^2^ culture flasks) following the manufacturer’s instructions (RNeasy Micro Kit, Qiagen). Subsequently, 300 ng of total RNA for each sample were used for the reverse transcription (Qiagen Qiatect Reverse Transcription, Switzerland) as described before^19^. The synthetized cDNAs were used for reverse transcription PCR amplification (RT-PCR) with the primers listed in Table 1. *Cypa* - coding for peptidylprolyl isomerase A, also known as cyclophylin A - was used as reference gene due to its reported stability as a housekeeping gene^27^. Primers sequences for *PPARGC1A* (peroxisome proliferator-activated receptor-gamma coactivator), *NRF1* (nuclear respiratory factor 1), *Tfam* (transcription factor A, mitochondrial), *COX1*, *COX4/1* and *Hprt* (Hypoxanthine Phosphoribosyltransferase 1) genes were obtained from^28^. Primers for *Bdnf* (Brain-derived neurotrophic factor), *Ngf* (Nerve growth factor) and *Ntrk2* (Neurotrophic receptor tyrosine kinase 2) are listed in Table 1. RT-qPCR was carried out using the universal 2X KAPA SYBR FAST qPCR Master Mix (Axonlab AG, Mont-sur-Lausanne, Switzerland). Gene expression quantitation was carried out in a DNA thermal cycler (Corbett Rotor gene 6000, QIAGEN Instruments AG, Hombrechtikon, Switzerland), according to a protocol described earlier^24,25^. For the semi-quantitative analyses of *Pvalb* transcript levels shown in Fig. 1, the thermocycler was stopped after 25, 30 and 35 cycles, 4 µl of the PCR product was removed from the RT-PCR reaction tubes and loaded on 2% agarose gels for electrophoresis. For the RT-qPCR reactions of mitochondrial and other genes, the following two-step protocol was applied: an initial denaturation step of 95 °C for 3 min followed by 30 cycles of: denaturation at 95 °C for 3 s and annealing/extension/data acquisition at 62 °C for 20 s. Normalization of mRNA levels was performed as described in^29^.

**Table 1:** Primers used for RT-qPCR and/or RT-PCR.

Gene Accession	Gene symbol	Gene description	FW Primer	RV Primer	Size product
XM_006241929	Pvalb	parvalbumin	ATGTCGATGACAGACTTGCTC	TTAGCTTTCGGCCACCAGAGT	343 bp
XM_006250801.3	Cypa	cyclophilin A	TATCTGCACTGCCAAGACTGAGTG	CTTCTTGCTGGTCTTGCCATTCC	126 bp
NM_001277055.1	NGF	Nerve Growth Factor	AGTGTGTGGGTTGGAGATAAG	GCATCCACTCTCTACAGGATTC	150 bp
NM_001270632.1	BDNF	Brain Derived Neurotrophic Factor	GGTCGATTAGGTGGCTTCATAG	CGAACAGAAACAGAGGAGAGATT	149 bp
AY_265419.1	Ntrk2	Neurotrophic receptor tyrosine kinase 2	GAAGGGAAGTCTGTGACCATTT	CTGTGTGTGGCTTGTTTCATTC	114 bp

### Western blot assay

Cells were seeded in 75 cm^2^ flasks (TPP, Trasadingen, Switzerland) and harvested at 80–90% of confluence. Proteins were extracted from PBS-washed cell pellets and ultrasonicated in TE buffer (with protease inhibitors), centrifuged at 13,000 rpm and the supernatant was collected. Protein concentration was determined with the Bradford assay. Proteins (50 µg) were separated on 12% polyacrylamide SDS gels and transferred onto nitrocellulose membranes. Loading was controlled using Ponceau S staining and membranes were blocked with 5% milk in 0.1% Tween 20 in TBS for 1 h at room temperature and incubated overnight at 4 °C with rabbit polyclonal antibody PV-25 (Swant, Marly, Switzerland) at 1:5,000, or mouse monoclonal COX I antibody (ThermoFisher, Switzerland) at 1:1,000, respectively. The PV-25 antibody has been validated for Western blot analysis in our previous study^20^. Horseradish peroxidase (HRP)-conjugated goat anti-rabbit antibody or goat anti-mouse antibody (Sigma-Aldrich, Buchs, Switzerland) were added at a dilution of 1:10,000 for 2 h at room temperature. The HRP substrate (Millipore, Luminata Forte) was applied to the membrane for 1 min and membranes were imaged on the Western Blot reader FluorChem E System (Bucher Biotec, Basel, Switzerland). The GAPDH signal (polyclonal rabbit, Sigma-Aldrich, Buchs, Switzerland, #G9545, dilution for Western blot: 1:10,000) was used as loading control.

### Growth of transduced CG4 cells

C-CG4, GFP-CG4, PV-CG4 and shPV-CG4 cells were seeded in 24-well plates (20,000 cells/well in proliferation medium and 40,000 cells/well in differentiation medium) and growth curves were established using the Incucyte Live Cell Imaging System (EssenBioscience, USA). The slopes of the growth curves were determined during the exponential phase of proliferation. In another set of experiments, we mixed populations of GFP-transduced CG4 cells with PV-expressing or shPvalb-expressing CG4 cells in a ratio of 1:1, and cultured them for several weeks. We measured the proportion of GFP+ cells after each passage by fluorescence activated cell sorting (Accuri C6 Plus, BD Biosciences, San Jose, CA, USA) using the FlowJo analysis platform (LLC, Ashland, Oregon, USA) for single-cell flow cytometry analysis. The experiments were performed in triplicates.

### Wound closure and cell migration assay

CG4 cells were seeded in ImageLock 96-well microplates (Essen Bioscience) (20,000 cells/well in proliferation medium and 20,000 cells/well in differentiation medium) and subjected to the scratch wound assay on the next day. A scratch wound was carried out by a 96-pin woundmaking tool (IncuCyte® WoundMaker 96, Essen Bioscience, USA) and cells were imaged every 60 min by the IncuCyte system. This device created homogeneous, 700–800 µm wide scratch wounds in CG4 cell monolayers. Scratch wound closure was analyzed by the IncuCyte software. Different parameters such as wound width, relative wound density and wound confluence were plotted over time. We chose the relative wound density as the parameter that varied the least between individual experiments and the areas under the curve (AUC) were calculated and normalized to the control, and then averaged per condition using GraphPad software. Single cell tracking was performed on video sequences by ImageJ using the Manual Tracking plugin. Cell velocity (µm/min) and travelled distance (µm) were calculated and averaged per condition. The experiments were performed in triplicates.

### Measurement of cellular process’ length

For evaluation of the cellular process outgrowth in CG4 cells, automatically acquired real-time images from the IncuCyte Imaging system were further analyzed using Fiji software. Three different time points were selected for further analysis; i.e., 6, 12, and 24 h after seeding. After this time, cells formed a highly connected network, therefore it was impossible to track single cells with all of its processes. The length of cellular processes was measured using the Fiji software after setting the measurement scale. Criteria for the evaluation of cellular processes outgrowth were adapted as described previously^30,31^, i.e. CG4 cells with at least one cellular process greater than the diameter of a cell body was considered as differentiated. Average process’ length was evaluated by measuring the total length of all processes per cell and divided by the number of processes in the same cell. Only wells with a confluence of 10–20% were analyzed. The experiments were performed in triplicates.

### Confocal microscopy and 3D reconstruction

The differences between CG4 cells were qualitatively and quantitatively examined using live-cell confocal imaging. The method is in line with previous studies^23,25,32^ with some modifications. Briefly, CG4 cells seeded onto glass-bottom dishes (MatTek Corp, Ashland, USA) were cultivated in normal or differentiating medium for 3 days. Before experiments, CG4 cells were loaded for 30 min with Calcein-AM (1 µM) and MitoTrackerRed CMXRos (500 nM) to detect volume changes in cells or mitochondria, respectively. Nuclei were stained with Hoechst 33342 (1 µM) for the last 5 min of the Calcein-AM loading. Cells were washed carefully and visualized in DPBS medium using a Leica TCS SP5 confocal microscope with a 40x oil-immersion objective (1.3 NA, Plan APO). Images were acquired along the z-axis with a 0.34 µm step interval. Morphometric analyses were done automatically by the Imaris 9.1.1 software (Bitplane AG, Switzerland), using the “ImarisCell” module, specifically designed for the analysis of 3D images of cells, nuclei and multi-class, multi-sized vesicles. The fluorescent threshold for the green (cytoplasm), blue (nucleus), and red (mitochondria) signals was kept constant between different groups. At least 80 cells (only cells with a single nucleus) were analyzed in each group. In addition, the mitochondrial length and diameter in the optical plane were measured from maximal xy projections in Imaris software using an “Imaris Measurement Pro” module.

### Measurement of mitochondria in cellular processes

To evaluate mitochondrial density in distal cellular processes, confocal images were analyzed in Imaris software and measured using the “Imaris MeasurementPro” module. First, the length of each mitochondrion in a cellular process was marked and shown as an average value of that process. Then the length of cellular processes was examined as well. For the calculation of mitochondrial density, the total length of mitochondria in one process was divided by the process’ total length (total mitochondrial length in one process/length of the same process). At least 80 cells were analyzed in each group (with at least 200–400 processes). Data are shown as averaged values in µm. The experiments were performed in triplicates.

### Measurement of acute phototoxicity-induced ROS production by dihydroethidium (DHE)

Undifferentiated and differentiated cells were plated in pre-coated glass-bottom plates (50,000 cells/dish) and cultured for 24 h prior to experiments. We induced reactive oxygen species (ROS) production in the cells with pulses of UV light exposure by a Leica TCS SP5 confocal laser-scanning microscope (imaging with a 405-nm UV laser at 60% AOTF for 1 s followed by 9 s pause, for a total of 10 min by a 20x objective at 3x zoom, thus exposing an area of 258 µm × 258 µm). Oxidative stress was assessed by the live superoxide indicator dihydroethidium (DHE, Thermo Fisher Scientific, Reinach, Switzerland). DHE exhibits blue fluorescence in the cytosol until oxidized, where it then intercalates in the cell’s DNA, staining its nucleus bright fluorescent red. 24 h after plating, cells were gently rinsed with pre-warmed PBS and incubated for 25 min at 37 °C in DHE solution (2 µM in Opti-MEM). Next, cells were gently rinsed with pre-warmed PBS and immediately used for confocal imaging. To assess the fluorescent intensity of the cells, the Leica Application Suite Advanced Fluorescence software (LAS-AF) from Leica Microsystems (Leica, Germany) was used. Each cell was identified as a region of interest (ROI) after background subtraction, the mean fluorescent intensities in the blue and red channels were plotted over time, and the values of start (0 min) and the end of experiment (10 min) were averaged from the first three (0–20 s), and last three time points (580–600 s), respectively. We then calculated the delta (end–start) of the DHE fluorescence intensity in the red channel. The experiments were performed in triplicates. Statistical analysis (one-way ANOVA or Kruskal-Wallis test) was performed with Graph Pad Prism 7 software upon removal of outliers (values significantly different from the other values within the group) using ESD (extreme studentized deviate) method in Graph Pad software.

### Hypoxia

CG4 cells were seeded in 96-well plates (2,000 cells/well in proliferation medium and 4,000 cells/well in differentiation medium) and maintained in culture for 3 days in a hypoxia incubator (1% O_2_), while control plates were maintained in culture for 3 days under normal culture conditions. RT-PCR and Western blot assays were performed as described above. CG4 cells were seeded in 75 cm^2^ flasks (TPP, Trasadingen, Switzerland) and harvested 3 days after incubation in normoxic or hypoxic condition, respectively. Cell viability was assessed with the MTT assay detailed in the next section. The experiments were performed in triplicates.

### Proliferation and viability assay

CG4 cells were seeded in 96-well plates (2,000 cells/well in proliferation medium and 4,000 cells/well in differentiation medium) and maintained in culture until 80–90% confluence. The tetrazolium dye MTT 3-(4,5-dimethylthiazol-2-yl)-2,5-diphenyltetrazolium bromide was added to the culture medium at 0.5 mg/ml and incubated at 37 °C for 3 h. The formazan crystals were dissolved in DMSO and the plate was agitated for 30 min at room temperature, and absorbance at 590 nm was measured by a spectrophotometric multiwell plate reader (Victor X3 by Perkin Elmer, Waltham, MA, USA), followed by normalization of the absorbance values to the control.

### Statistics

All data are presented as mean ± standard error of the mean (SEM), from at least three independent experiments. For statistical comparisons and graph generation the GraphPad Prism 7.04 software (GraphPad Software, San Diego, USA) was employed. For the normality test, the D’Agostino–Pearson omnibus test was applied. For normally distributed data one-way ANOVA followed by the Tukey post-test was used. Otherwise, Kruskal–Wallis tests followed by Dunn’s test was utilized to compare differences between experimental groups. The P values smaller than 0.05 were assigned as significant; P < 0.05*, P < 0.01**, P < 0.001***, P < 0.0001****.

## Results

### Initial characterization of genetically modified CG4 cells

The OPC line CG4 was used as a model system to investigate the role of PV in the course of I) process formation, II) ROS production and III) protection against hypoxia. Initial experiments were aimed to characterize the effects of PV on CG4 cell morphology, cell growth/proliferation and cell mobility/invasiveness. Cells maintained in B104-conditioned medium remained in an undifferentiated state characterized by a bipolar, fusiform cell shape with long cellular processes (Fig. 1A); cells grown in differentiating medium resulting in a OLG-like phenotype showed a characteristic morphology with multiple elongated, branching processes and rounded cell bodies (Fig. 1B). Analyses of *Pvalb* transcripts revealed the presence of low amounts of *Pvalb* mRNA in control (C-) undifferentiated and differentiated CG4 cells. A positive but rather weak signal in the semi-quantitative RT-PCR was obtained after 30 cycles (Fig. 1C,D; complete and unprocessed images are shown in Suppl. Fig. S1), indicative of rather low endogenous *Pvalb* transcript levels. RT-qPCR analyses revealed *Pvalb* mRNA levels in undifferentiated and differentiated C- and GFP-CG4 cells to be rather similar (Fig. 1C,D; lower parts); in shPV-CG4 cells, the *Pvalb* transcript signals were not above background level, revealing efficient shRNA-mediated *Pvalb* mRNA degradation that had led previously to near complete PV down-regulation in PV-MDCK cells using the same shPvalb construct [25]. Analyses of PV protein expression levels in CG4 cell lysates from both undifferentiated and differentiated cells revealed very low-to-none endogenous expression in C-CG4 cells (Fig. 1E,F). With lentiviral techniques we successfully transduced CG4 cells with a lentivirus (LV) leading to PV overexpression (PV-CG4 cells), a LV expressing an shRNA against the *Pvalb* gene (shPV-CG4 cells) or a LV producing GFP, the latter serving as a transduction control. *Pvalb* mRNA levels fell below the detection level in shPV-GC4 cells, while in cells transduced with the PV-LV, a strong RT-PCR signal was evident (Fig. 1C,D). The presence of PV in the overexpressing CG4 cells was confirmed by Western Blot analyses (Fig. 1E,F). Results on *Pvalb* mRNA and PV protein levels were identical in undifferentiated and differentiated CG4 cells, indicating that the differentiation status had no effect on *Pvalb* mRNA and PV levels (Fig. 1C–F). Cells overexpressing GFP (GFP-CG4) cells were used as a transduction control; the efficient upregulation of GFP was confirmed by fluorescence microscopy (Suppl. Fig. S2A,B) and by FACS analysis (Suppl. Fig. S2C). On low magnification images no obvious differences in the cell morphology between C-, GFP-, shPV- and PV-CG4 cells were observed, neither in undifferentiated (Fig. 1G) nor in differentiated (Fig. 1H) cells. Since endogenous PV levels in C-CG4 cells were below the detection limit for Western blot analyses and no striking differences were observed between shPV- and either C- or GFP-CG4 cells, further experiments were essentially carried out with PV-overexpressing PV-CG4 cells and compared to either C-CG4 or GFP-CG4 cells, the latter serving as a control to possibly detect changes caused by LV transduction. In experiments where the shPV-CG4 cells were included, in none of these experiments significant differences compared to C-CG4 or GFP-CG4 cells were observed; results are presented in Supplemental Figs. S3 and S5 and for clarity were omitted in the main figures.

### PV does not affect proliferation of CG4 cells

**Figure 2:**
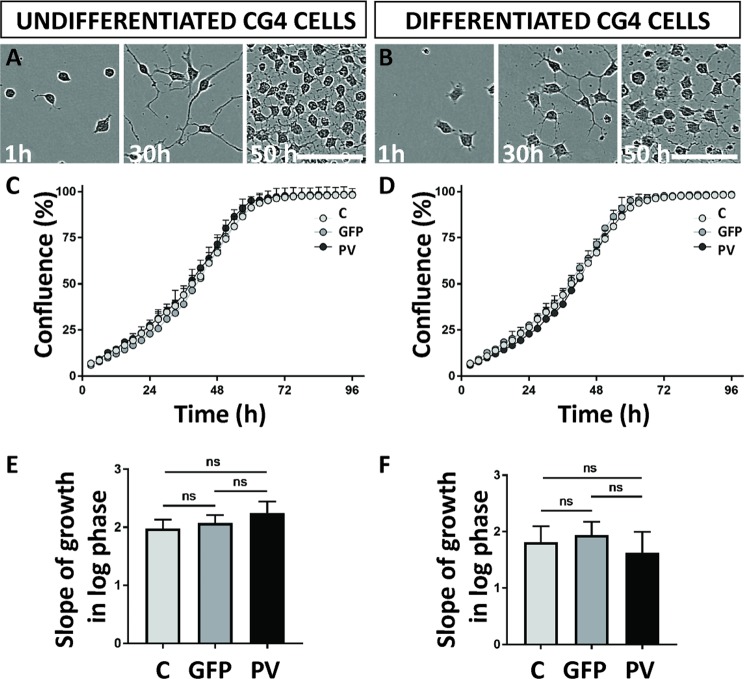
Growth properties are unaffected after PV upregulation. Confluence analysis of undifferentiated CG4 cells (**A**) or differentiated CG4 cells (**B**) with representative images (C-CG4 cells) taken by the IncuCyte system 1 h, 30 h and 50 h after plating showed no significant alterations in the growth curve slopes of C-, GFP- and PV-CG4 cells in the undifferentiated state (**C**,**E**) or in the differentiated state (**D**,**F**).

Cell proliferation of transduced CG4 cells was monitored with a live-cell imaging system. Growth curves were established based on cell confluence (Fig. 2A–D) and the slopes of the growth curves were determined during the exponential (log) phase of proliferation (Fig. 2E,F). Among undifferentiated CG4 cells (C-, GFP-, and PV-), no differences in proliferation rates were observed: control cells displayed a slope (the rate of change along the regression line of the log phase) of 1.981 ± 0.152 (all values are mean ± standard error of the mean (SEM), GFP-CG4 cells of 2.075 ± 0.137 and PV-CG4 cells of 2.244 ± 0.200 (*p* = 0.6674, one-way ANOVA). No differences were also detected in the differentiated cells: C-CG4 cells had a slope of 1.813 ± 0,281, GFP-CG4 cells one of 1.942 ± 0.235 and PV-CG4 cells a slope of 1.630 ± 0.368 (*p* = 0.8961, one-way ANOVA). Since the differences were minimal as observed in the proliferation experiments lasting for 96 h, we additionally ascertained that all cells show indeed unaltered proliferation properties. With this aim, we mixed populations of GFP-CG4 and either PV-CG4 or C-CG4 cells at a ratio of 1:1, and determined the proportion of GFP-positive (GFP+) cells after each passage by FACS analysis for several weeks. Even after 8 passages, the ratio between green and non-green cells remained constant precluding even small differences in proliferation rates of GFP-CG4 and C-CG4 cells, as well as between GFP- and PV-CG4 cells. This was the case for CG4 cells in their undifferentiated and differentiated states (Suppl. Fig. S2C).

### Minor effect of PV expression on CG4 cell migration and cell velocity

**Figure 3:**
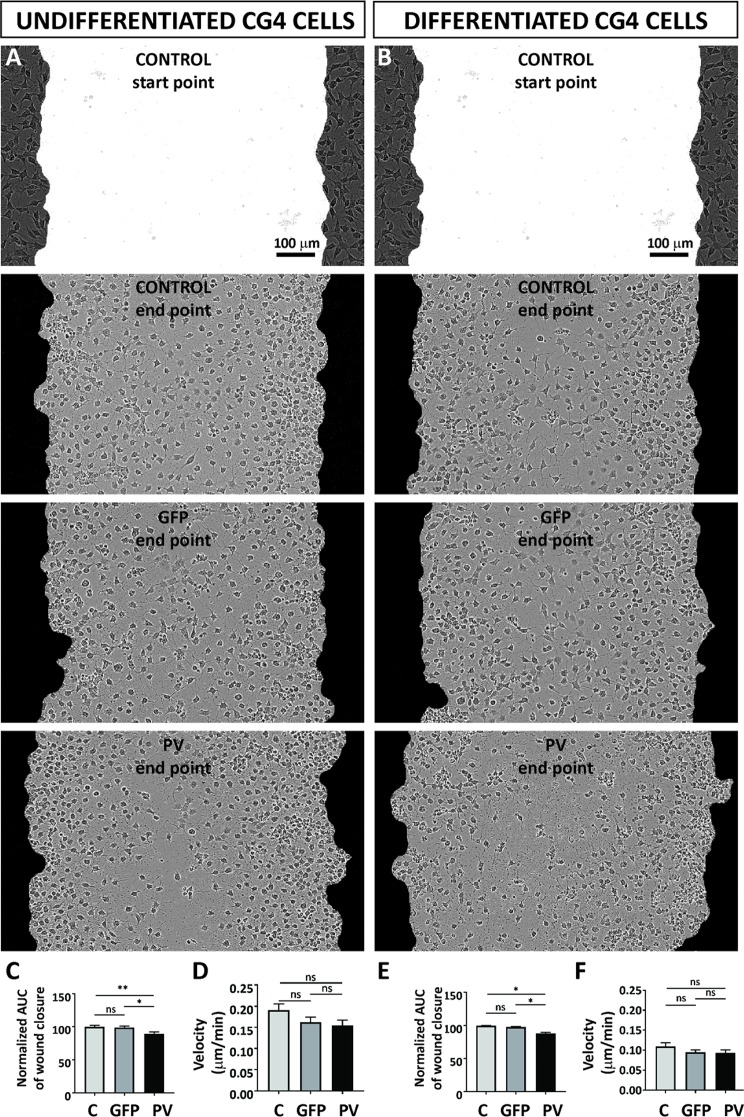
PV expression affected “wound closure” capacity as assessed by a scratch wound assay using undifferentiated C-, GFP- and PV-CG4 cells (**A**) and the same differentiated CG4 cell lines (**B**). The wound closure capacity was evaluated by calculating the AUC of the relative wound density plotted over time, which was normalized to the control (C-CG4 cells) and averaged per condition. In the undifferentiated OPC state PV-CG4 cells displayed slightly decreased AUC values (relative wound density over time) when compared to C-CG4 cells (**C**), suggesting a reduced wound closure capacity in PV-overexpressing CG4 cells in the undifferentiated state. Similar differences were also present in CG4 cells in the differentiated state (**E**). Single-cell tracking from video sequences revealed no significant differences in the cell velocity (µm/min) of CG4 cells, in both the undifferentiated OPC-like (**D**) and differentiated OLG-like states (**F**). Error bars represent SEM (ns: not significant; *p* value > 0.05, *p < 0.05, **p < 0.001).

Cell migration and velocity of the different CG4 cell lines was tested in the “wound healing” or “scratch“ assay. In a confluent CG4 cell layer a scratch of approximately 700–800 µm was introduced (Fig. 3; upper figures, marked as “start point”) and the closing of the gap by cell repopulation was monitored in real time (Fig. 3A,B). Larger cell-devoid regions in the middle of the scratch were observed with PV-CG4 cells. The relative wound density values normalized to control C-CG4 cells were determined for GFP- and PV-CG4 cells, calculated as AUC. Undifferentiated PV-CG4 cells showed a slightly reduced wound closure capacity compared to C-CG4 cells (89.23% ± 2.95%; *p* = 0.0072 PV- *vs*. C-), while no differences existed between C-CG4 and GFP-CG4 cells (96.68% ± 2.31%; *p* = 0.9240) (Fig. 3C). In all 3 groups of differentiated CG4 cells the wound closure capacity showed a similar trend (97.84% ± 1.05% for GFP-CG4 and 88.46% ± 1.72% for PV-CG4, *p* < 0.0140, Fig. 3E). We summoned that the differences in wound closure capacity might be the result of altered cell velocity. Although velocity of PV-CG4 cells was found to be reduced, qualitatively to a similar extent as the wound closure capacity, the differences were not significant (Fig. 3D,F): 0.1906 ± 0.015 µm/min for C-CG4 cells, 0.1568 ± 0.012 µm/min for GFP-CG4 cells and 0.154 ± 0.012 µm/min for PV-CG4 cells (Fig. 3D). For differentiated cells, the bar graph of velocities (Fig. 3F) closely resembled the wound closure capacity indicating that PV had no effect on migration/mobility (velocity) in differentiated CG4 cells: 0.1088 ± 0.009 µm/min for CG4 control cells, 0.09006 ± 0.006 µm/min for GFP-CG4 cells and 0.0925 ± 0.008 µm/min for PV-CG4 cells). Thus, besides unchanged proliferation rates, PV overexpression had no substantial effect on cell migration and cell velocity most clearly seen in differentiated CG4 cells.

### Increased PV expression decreases mitochondrial volume in undifferentiated and differentiated CG4 cells

As we had previously observed robust effects of PV on the morphology of Madin-Darby canine kidney (MDCK) epithelial cells^25^, we tested whether (similar) changes also occurred in cells of glial lineage. For this, the cytosolic volume was stained with Calcein-AM (green), nuclei with Hoechst 33342 (blue) as described previously^25,32^ and mitochondria were stained with MitoTrackerRed CMXRos (red) allowing for 3D-volume rendering analysis. Unlike the rather homogenous MDCK cells, in general, CG4 cells showed a rather high variability in cell morphology and also in the length of cellular processes. Moreover, the distribution of mitochondria within the cell body, as well as in cellular processes, i.e. in distal segments looked quite different and was thus analyzed separately, as carried out in a previous study in the soma and dendrites of Purkinje cells^23^.

**Figure 4:**
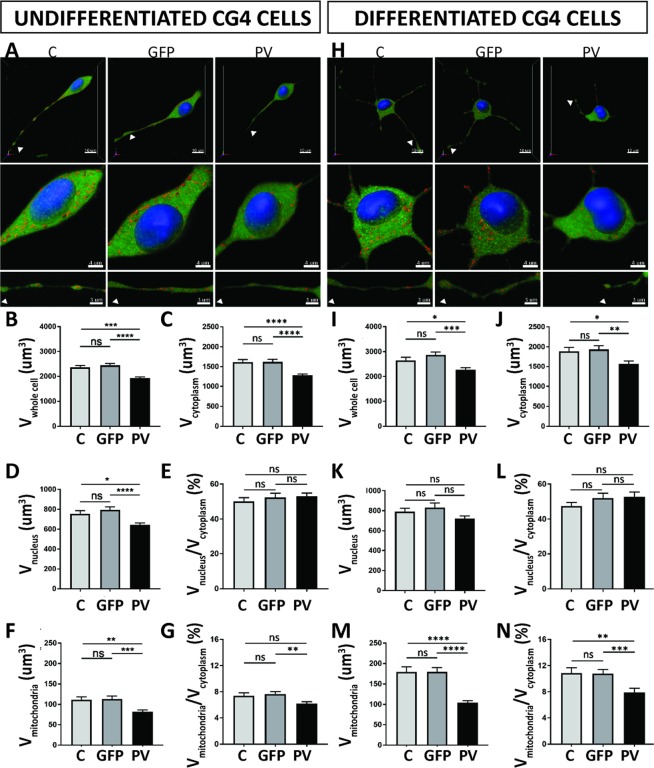
PV upregulation alters the morphology and soma mitochondria volume of CG4 cells. Cells were loaded with MitoTrackerRed CMXRos (Red), Hoechst (blue) and Calcein-AM (green) and z-stacks were imaged by confocal laser microscopy (**A**,**H**) to determine volumes of soma mitochondria, nuclei and soma cytoplasm, respectively. Quantification of total cell (soma) volume (**B**,**I**), cytoplasmic (soma) volume (**C**,**J**), volume of nucleus (**D**,**K**), ratio of nucleus/soma cytoplasm (**E**,**L**), soma mitochondria volume (**F**,**M**) and ratio of soma mitochondria/ soma cytoplasm (**G**,**N**) was performed by the Imaris software. The soma mitochondria volume and moreover the ratio of mitochondria/cytoplasm were significantly lower in PV-CG4 cells when compared to C-CG4 (control) and GFP-CG4 cells. Error bars represent SEM. (ns: not significant; *p < 0.05, **p < 0.01, ***p < 0.001, ****p < 0.0001.

Compared to undifferentiated C-CG4 cells (Fig. 4A), the whole-cell volume (somata only) of PV-CG4 cells was approximately 19% smaller (Fig. 4B; *p* = 0.0352), the soma cytoplasmic volume (Fig. 4C), as well as the volume of nuclei (Fig. 4D) was decreased to approximately the same extent. The soma cytoplasmic volume was 1616 µm^3^ in C-CG4 cells and 1280 µm^3^ in PV-CG4 cells. As in MDCK cells the ratio of nuclear/soma cytoplasmic volume (Fig. 4E) was unchanged signifying that PV overexpression similarly decreased the volumes of the soma cytoplasm and nuclei (Fig. 4C,D). PV overexpression caused a 27% decrease in the relative soma mitochondrial volume in the cell bodies from 111.4 µm^3^ in C-CG4 cells to 81.57 µm^3^ in PV-CG4 cells (Fig. 4F). The reduction in soma mitochondrial volume in PV-CG4 cells was more pronounced (over-proportional) compared to the general PV-induced “cell shrinking”, i.e. the normalized ratio soma mitochondria/cytoplasm volume was still smaller in PV-CG4 cells (Fig. 4G). This hints towards the possibility of reduced energy production in PV-CG4 cells and moreover PV overexpression mostly decreasing mitochondria volume, which in turn might be a likely cause for the globally decreased size of PV-CG4 cells. Globally results were identical in differentiated CG4 cells (Fig. 4H) and the effect size between cells expressing PV and C-CG4 cells was slightly larger. The whole-cell volume was decreased by 14% (Fig. 4I; *p* = 0.0438). While there were proportional changes in the volume of soma cytoplasm (Fig. 4J) and nuclei (Fig. 4K) between C-CG4 and PV-CG4 cells, PV upregulation did not affect the ratio of nucleus/cytoplasm volume (Fig. 4L). Also in differentiated CG4 cells, the soma mitochondrial volume (Fig. 4M) and the ratio soma mitochondria/soma cytoplasmic volume (Fig. 4N) was significantly decreased. The relative volume of soma mitochondria was decreased by 42% (Fig. 4M; *p* < 0.0001) and the ratio mitochondria/cytoplasm was 34% smaller (Fig. 4N). Of note, no differences were observed in any of the above parameters including soma mitochondria volume (as shown in Fig. 4B–N) between C-CG4 and shPV-CG4 cells (Suppl. Fig. S3) indicating that the small amounts of *Pvalb* mRNA present in C-CG4 cells had, most importantly, no measurable effect on soma mitochondria volume. These findings confirm a reciprocal effect of PV on soma (whole-cell) volume and an even stronger one on soma mitochondria volume also in CG4 cells, irrespective whether they were differentiated or not. Effects of PV on mitochondria density and localization in CG4 cell processes are described below.

### Decreases in mitochondrial density in PV-CG4 cells is reflected by a decrease in proteins implicated in mitochondria function and biogenesis

**Figure 5:**
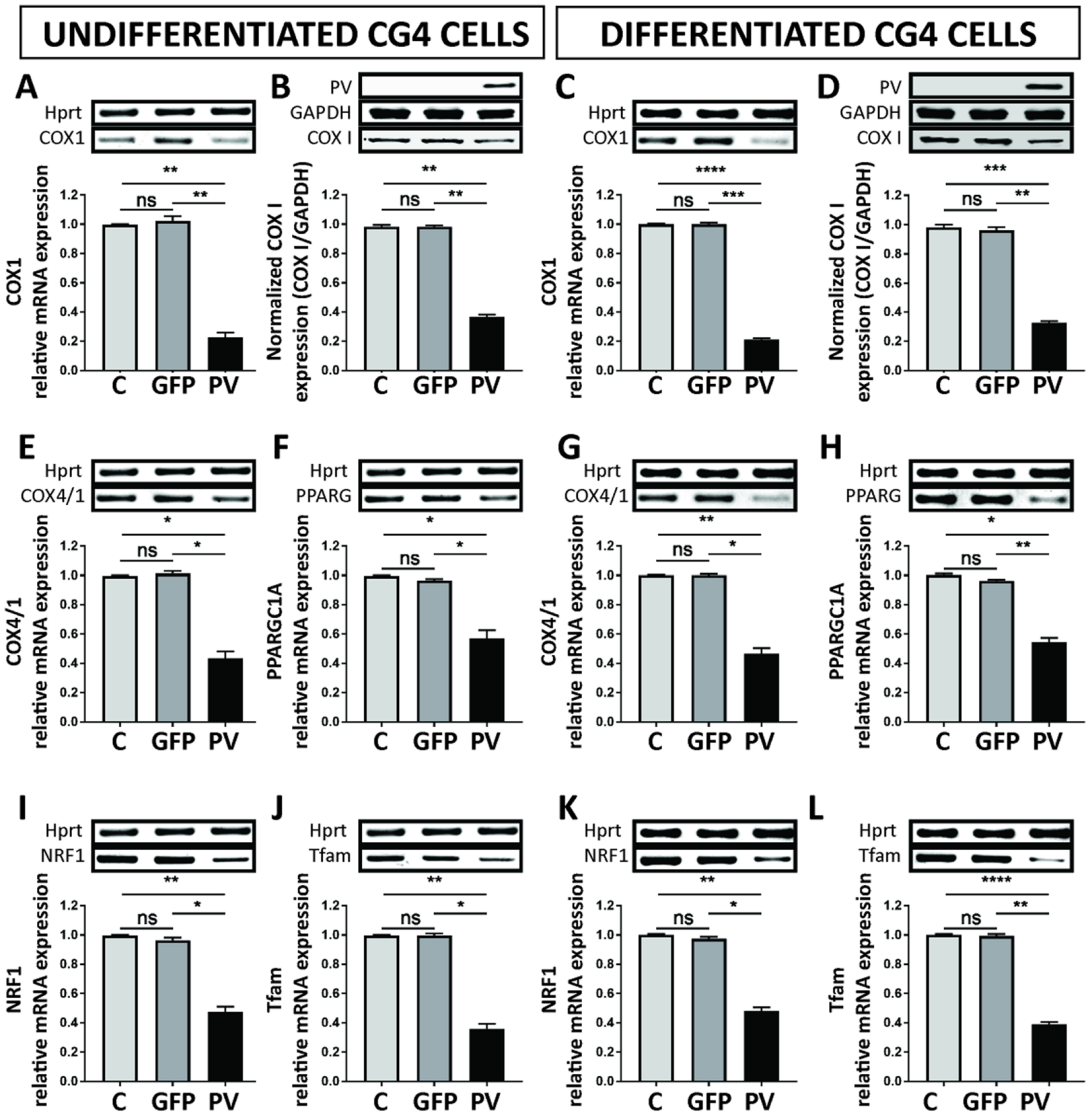
PV upregulation alters the expression of mRNA or proteins implicated in mitochondria functions and biogenesis. mRNA expression levels of *COX1* (**A**,**C**) and protein expression levels of COX I (**B**,**D**) are shown, as well as mRNA expression levels of *COX4/1* (**E**,**G**), *PPARGC1A* (**F**,**H**), *NRF1* (**I**,**K**) *Tfam* (**J**,**L**). The signal for *Hprt or* GAPDH was used for the normalization of mRNA or protein expression, respectively. Representative image of gels are shown above each graph. (n.s. = not significant, *p < 0.05, **p < 0.01, ***p < 0.001, ****p < 0.0001).

Since morphological changes in cell bodies with respect to reduced relative mitochondrial volume were similar in undifferentiated and differentiated PV-CG4 cells, we expected to see also similar changes with respect to mitochondrial transcripts and proteins implicated in mitochondrial function and mitochondria biogenesis. As a first observation all changes reported below are equally valid for undifferentiated and differentiated CG4 cells (Fig. 5; complete gels are presented in Suppl. Fig. S4). Cytochrome c oxidase subunit I (COX I; *COX1*) is encoded by the mitochondrial DNA, while the cytochrome oxidase assembly protein (COX IV) complex located in the inner mitochondrial inner membrane is encoded by the nuclear DNA. Levels of *COX1* (Fig. 5A,C) and *COX4/1* (Fig. 5E,G) transcripts were significantly reduced in PV-CG4 cells compared to both C- and GFP-CG4 cells and moreover also protein levels of COX I were decreased to the same extent (Fig. 5B,D) confirming the data from mitochondria morphology. Transcript levels of proteins implicated in mitochondria biogenesis were subsequently analyzed. The transcript (*PPARGC1A*) encoding the peroxisome proliferator-activated receptor-gamma coactivator (PGC-1α), a master regulator of mitochondria biogenesis and of cellular energy metabolism, was strongly decreased in PV-CG4 cells (Fig. 5F,H), as were levels of the downstream PGC-1α target nuclear respiratory factor NRF-1 (Fig. 5I,K), which together with NRF-2 regulates the expression of both mitochondrial and nuclear genes encoding respiratory chain subunits and other proteins that are required for mitochondrial function^33^. NRF-1 is also implicated in the regulation of neurite outgrowth^34^. Also *Tfam* mRNA levels encoding the transcription factor A mitochondrial protein (Tfam) important for mitochondrial DNA replication were decreased (Fig. 5J,L). In conclusion, transcript levels of proteins implicated in mitochondria biogenesis were strongly downregulated in PV-CG4 cells, likely resulting in the lower mitochondrial volume observed in these cells.

### Mitochondrial density and position of mitochondria in distal cellular processes are affected by PV expression

**Figure 6:**
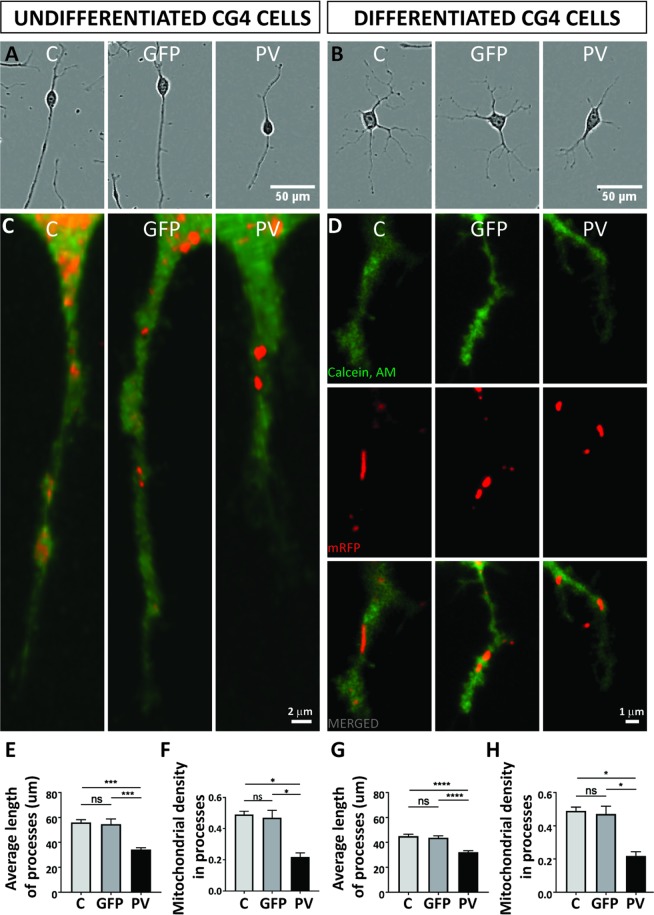
PV overexpression affects mitochondrial density and position of mitochondria in distal cellular processes. Representative CG4 cells are depicted from IncuCyte images (**A**,**B**). Mitochondria were visualized by mitochondria-targeted red fluorescent protein (red) and cellular processes were visualized by Calcein-AM (green). Representative images are shown for C-, GFP- and PV-CG4 cells (**C**,**D**). In (**D**) separate images for cytoplasm (green, upper), mitochondria (red, middle) and merged images (lower) are shown. In addition, average length of processes (**E**,**G**) and mitochondrial density in the processes (**F**,**H**) was analyzed. Cellular process length of CG4 cells was evaluated by automatically acquired real-time images by the IncuCyte Imaging system. Single cells were analyzed and the length of cellular processes was measured. Average process’ length was evaluated by measuring total length of all processes per cell and dividing this number by the number of processes in the same cell. Error bars represent SEM, *p < 0.05, **p < 0.01, ***p < 0.001, ****p < 0.0001.

Growth and elongation of cell processes necessitates a significant amount of energy^6^ and to ensure these energy requirements the precise mitochondrial position is important, especially at active growth sites^28^. Processes extending in a bipolar way in undifferentiated CG4 cells were on average considerably shorter in PV-overexpressing PV-CG4 cells determined 24 h after plating (Fig. 6A). The same hold also true for processes in differentiated PV-CG4 cells that radially extend their processes (Fig. 6B). In order to ascertain whether these length differences were associated with alterations in mitochondria in these cellular processes, the mitochondrial density was determined in processes of undifferentiated and differentiated CG4 cells according to the method reported in^35^. Data are shown as average values normalized per single cell. At least 80 cells from three different experiments were analyzed in each group. The mitochondrial density in processes was clearly lower in PV-CG4 cells in both undifferentiated (Fig. 6C) and differentiated cells with a mature oligodendrocyte-like phenotype (Fig. 6D). A comparison of the average length of processes and the mitochondrial density within processes reveled no differences between C- and shPV-CG4 cells, both in the undifferentiated and differentiated state (Suppl. Fig. S5). This is in line with the data on soma mitochondria volume, which was also not altered in cells with downregulated *Pvalb* mRNA (shPV-CG4) compared to C-CG4 cells as shown in Suppl. Fig. S3.

### PV-mediated changes in processes’ length in undifferentiated CG4 cells and altered processes’ branching in differentiated CG4 cells

The shorter average length of cellular processes in PV-CG4 cells (Fig. 6C,E; 34.29 ± 1.54 µm in PV-CG4 compared to 56.00 ± 2.20 µm in C-CG4 cells; *p* = 0.0001), as well as the decreased mitochondrial density in these processes (Fig. 6C,F, C-CG4 cells; 0.2664 ± 0.03 in PV-CG4 and 0.4258 ± 0.03 in control CG4 cells, *p* = 0.0173) are compatible with reduced energy production in the processes of PV-overexpressing CG4 cells. Of note the average length of individual mitochondria within CG4 cell processes was not different in cells with or without PV expression, whether in undifferentiated (C-CG4: 2.014 ± 0.059; GFP-CG4: 2.078 ± 0.063, PV-CG4: 1.997 ± 0.169; shPV: 1.962 ± 0.059; p > 0.05; values are mean ± SEM) or in differentiated CG4 cells (C-CG4: 2.077 ± 0.147; GFP-CG4: 2.112 ± 0.074, PV-CG4: 2.103 ± 0.121; shPV: 2.09 ± 0.084; p > 0.05). However, considerably less mitochondria were observed at the end of elongating processes and close to the growth cone, if PV was upregulated in PV-CG4 cells, (Figs. 4A and 6C) with enlarged part of processes and mitochondria (n > 100 processes in each group). Essentially identical results were obtained in CG4 cells grown in differentiating medium. Representative images are shown in Figs. 4H and 6D. The quantitative analyses are depicted in Fig. 6G,H.

**Figure 7:**
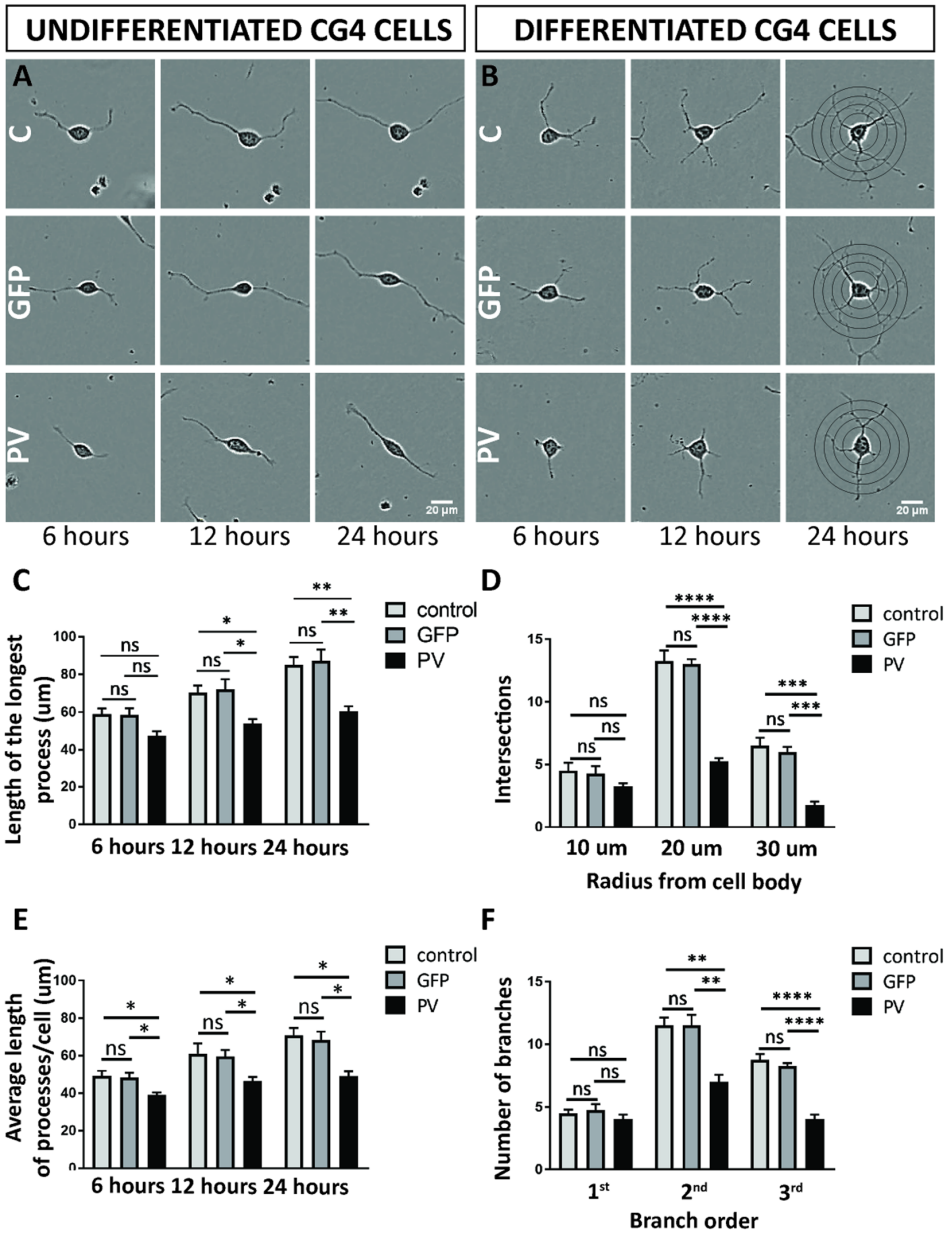
Length of processes in undifferentiated CG4 cells and Sholl analyses of process’ branching in differentiated CG4 cells. Representative undifferentiated (**A**) and differentiated (**B**) CG4 cells are depicted from IncuCyte images obtained at 6, 12 and 24 h after seeding. For the length of the longest process (**C**) and average length of processes/cell (**E**), all time points (6, 12, 24 h) are shown. Sholl analyses of process’ branching in differentiated CG4 cells is shown for 24 h after seeding (**D**,**F**). The number of intersections (**D**) was analyzed at a 10-, 20- and 30-µm radius from the cell body. Number of branches (**F**) was analyzed for first, second and third branch order (n.s. = not significant, *p < 0.05, **p < 0.01, ***p < 0.001, ****p < 0.0001).

To obtain better insight in the dynamics of process formation in CG4 cells with and without PV expression, we determined the length of the longest process and the average process length for a given cell 6, 12 and 24 h after plating in undifferentiated CG4 cells (Fig. 7A,C,E). Compared to either C-CG4 or GFP-CG4 cells, no significant differences with respect to the longest process were observed in PV-overexpressing cells at 6 h; however at 12 and 24 h the longest process was significantly shorter in PV-CG4 cells (Fig. 7C). Analyses of the average process length revealed shorter processes in PV- overexpressing cells at all investigated time points (Fig. 7E). Since differentiated CG4 cells are characterized by multiple, most often branched processes, most evident 24 h after plating, we performed Scholl analysis as typically used to determine neurite growth in nerve cells (Fig. 7B). At the shortest distance (10 µm) from the cell body the number of intersections was similar for all CG4 cell lines, while at 20 and 30 µm away from the cell body the number of intersections was significantly decreased in PV-CG4 cells Fig. 7D). Essentially identical findings were observed when looking at the number of branches: 1^st^ order branches were not different between cell lines, yet 2^nd^ and 3^rd^ order branches were clearly less numerous in PV-CG4 cells (Fig. 7F). Thus, in PV-CG4 cells characterized by a lower density of mitochondria in their processes, both process growth and process branching are clearly diminished compared to cells without PV expression (C-CG4 and GFP-CG4 cells). Since branching of oligodendrocytes during remyelination after injury is linked to brain-derived neurotrophic factor (BDNF) and/or nerve growth factor (NGF) signaling via neurotrophic tyrosine receptor kinase 2 (TrkB) receptor –in particular during the late part of repair– we investigated whether CG4 cells in their undifferentiated and/or differentiated states expressed measurable amounts of transcripts for the three molecules listed above. While a signal was obtained with total RNA isolated from rat brain, no evidence was detected for the presence of *Bdnf*, *Ngf* or *Ntrk2* mRNA in C-, GFP- and PV-CG4 cells, both in undifferentiated and differentiated cells (Suppl. Fig. S6).

### Increased PV expression levels lower ROS production in differentiated CG4 cells

**Figure 8:**
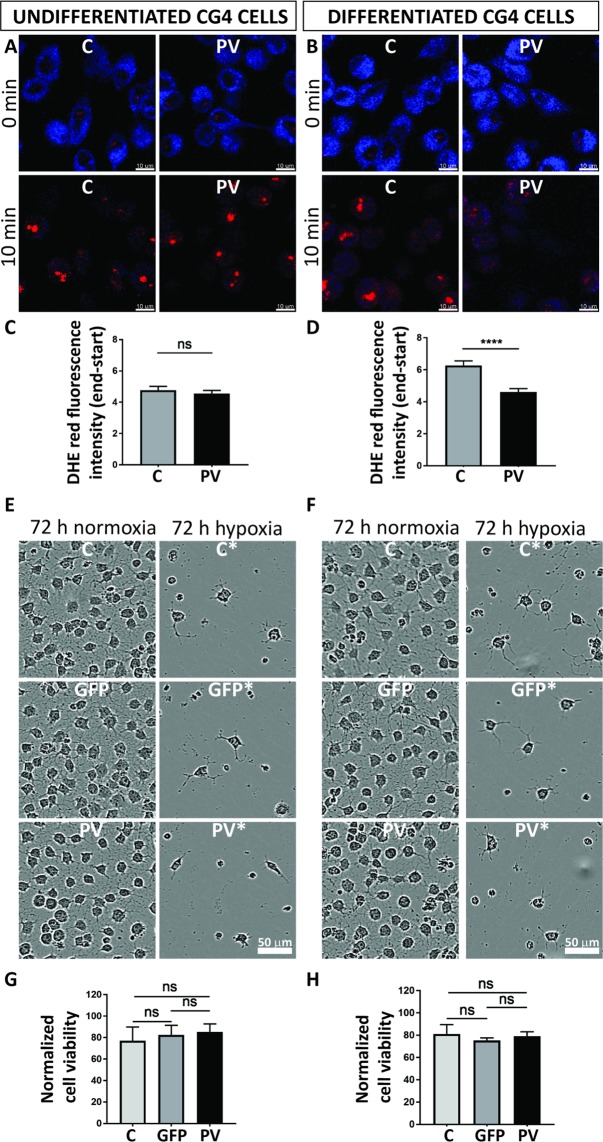
Measurement of phototoxicity-induced ROS production in CG4 cells. DHE-loaded control CG4 and PV-CG4 cells were exposed to UV-light pulses every 10 s for 10 min and imaged by confocal laser scanning microscope. DHE displays blue fluorescence in the cytosol until oxidized, then it intercalates in the DNA and stains its nucleus red (**A**,**B**). We then quantified the fluorescence intensity of each cell in the beginning and at the end of the UV-light exposure, and calculated the red delta ratio end–start (**C**,**D**). In the undifferentiated OPC state, we detected only a slight decrease of the superoxide levels in PV-CG4 cells in the red delta value, whereas there was a significant decrease of the superoxide levels in PV-CG4 cells in the differentiated OLG state (*p < 0.05, **p < 0.01, ***p < 0.001). Control CG4, GFP-CG4 and PV-CG4 cells were exposed to hypoxia (1% O_2_) for 72 h (**E**,**F**). Left: undifferentiated cells, right: differentiated cells. We measured cell viability in both control and hypoxic plates by MTT-assay, and normalized the viability after hypoxia to the untreated values. We detected no significant alterations when we examined the cell viability of C-CG4, GFP-CG4, PV-CG4 cells exposed to 72 h hypoxia, in either an undifferentiated OPC-like (**G**) or differentiated OLG-like state (**H**). Error bars represent SEM.

In reactive ependymal cells PV expression levels are inversely correlated with the degree of oxidative stress^19^ and furthermore PV-positive interneurons are highly susceptible to oxidative-stress-induced damage^36^. Thus, we hypothesized that PV expression levels in CG4 cells might have an effect on reactive oxygen species (ROS) production. Phototoxicity-induced ROS production induced by short UV light exposure was measured using the live-cell superoxide indicator DHE. DHE shows blue fluorescence in the cytoplasm in non-oxidizing conditions and exhibits red fluorescence localized to the nucleus upon oxidation. Unexposed healthy C-CG4 and PV-CG4 cells displayed a bright blue cytoplasm and weak red nuclear staining. UV interval exposure for 10 min induced oxidative stress as evidenced by the appearance of red nuclei and a decrease in the blue cytoplasmic staining (Fig. 8A,B). Due to the overlapping fluorescence emission of GFP and DHE, GFP-CG4 cells were excluded from these experiments. C-CG4 (n = 106 undifferentiated, n = 112 differentiated) and PV-CG4 cells (n = 157 undifferentiated, n = 103 differentiated) were analyzed. Fluorescence intensity values of the whole cells at the beginning and at the end of UV-light exposure were simultaneously recorded in the red and blue channels. Since the initial blue signals showed very high variability (large standard deviations), measurements of (Δ end - start) red/blue ratios were also highly variable. Thus, we quantified Δ red (end–start) values only. DHE red fluorescence signals were not different in UV-exposed undifferentiated C-CG4 and PV-CG4 cells CG4 cells (4.770 ± 0.245 vs. 4.562 ± 0.194, respectively; Fig. 8C). In differentiated C-CG4 the Δ red signal was clearly higher than in the PV-CG4 cells (6.263 ± 0.293 *vs*. 4.616 ± 0.2056, respectively; *p* = 0.0008 Kruskal-Wallis test; Fig. 8D). This indicates that ROS production induced by short-term UV exposure more strongly affected differentiated C-GC4 than the same cells in their undifferentiated state. Moreover in differentiated PV-CG4 cells ROS production was not increased indicating that PV overexpression in those cells prevented an increase in ROS production.

### Response to hypoxia is not affected by PV expression

Finally, we investigated whether PV expression in CG4 cells affected cell growth/viability in hypoxic conditions. Cells were maintained for 3 days either in a hypoxia incubator (1% O_2_) or in normal culture conditions (Fig. 8E,F). Cell viability/proliferation was assessed by the MTT assay and values were normalized to values of the corresponding normoxia-exposed CG4 cells. Undifferentiated post-hypoxic C-CG4 cells showed a decrease in viability to 77.1% ± 12.7%, GFP-CG4 cells to 82.4% ± 9.0%, and PV-CG4 cells to 85.3% ± 7.5% when compared to their corresponding untreated (normoxic) CG4 cell groups (Fig. 8G). The decrease was of similar magnitude in all groups indicating that PV overexpression had no measurable effect on hypoxia-impaired cell viability. The same hold true for differentiated post-hypoxic CG4 cells: C-CG4 cells (81.1% ± 8.5%), GFP-CG4 cells (75.3% ± 2.4%) and PV-CG4 cells (79.0% ± 4.1%), when compared to the corresponding normoxic CG4 cell groups (Fig. 8H). No significant differences existed between undifferentiated (*p* = 0.7626, one-way ANOVA) nor between differentiated CG4 groups (*p* = 0.7873, one-way ANOVA) indicating that elevated PV levels did not protect cells from the hypoxia-induced damage. To further strengthen this conclusion, we ascertained that not one of the two following possibilities occurred in CG4 cells subjected to hypoxia: I) Downregulation of PV in hypoxic PV-CG4 cells; II) Upregulation of PV in hypoxic C- and GFP-CG4 cells (characterized by low levels of *Pvalb* mRNA in basal (normoxic) conditions). RT-qPCR results indicated no changes in *Pvalb* mRNA levels caused by hypoxia, i.e. weak signals in C- and GFP-CG4 cells and a strong signal in PV-CG4 cells (Suppl. Fig. S7A,B). At the protein level, no signal for PV was detectable on Western blots using protein extracts from hypoxic C- and GFP-CG4 cells, as was the case for the same cells grown in normoxic conditions. In addition, there was no indication of a decrease in PV expression in PV-CG4 cells subjected to hypoxia (Suppl. Fig. S7C,D). Thus, changes in PV expression levels in either C- and GFP-CG4 or PV-CG4 cells caused by hypoxia can be excluded as the cause for the similar response to hypoxia of CG4 cells with or without PV expression.

## Discussion

### Antagonistic regulation of PV and mitochondria

Temporally and spatially precisely tuned Ca^2+^ signals are essential for many basic physiologic cellular functions including cell growth, metabolism, proliferation and cell mobility, just to name a few^37^. They are also highly relevant for complex cellular processes such as differentiation, programmed cell death or resistance mechanisms against neurodegeneration, oxidative stress and hypoxic-ischemic insults. In order to fulfill these many Ca^2+^-mediated tasks, cells make use of the components of the Ca^2+^-signaling toolkit including Ca^2+^ channels and pumps localized in the plasma membrane, as well as intracellular organelles, such as the endoplasmic reticulum, mitochondria and cytosolic Ca^2+^-binding proteins^38–40^. The network of Ca^2+^ signaling components, their interactions, as well as their regulation required for maintaining the physiological Ca^2+^ homeostasis has been termed the Ca^2+^ homeostasome^39^. One of the best-studied examples is the antagonistic regulation of the Ca^2+^-binding protein PV and mitochondria. Both represent cell-specific Ca^2+^ signal modulators implicated in determining the shape, amplitude and duration of Ca^2+^ signals in cells with high PV expression levels and/or a large mitochondrial volume^39,41^. The bidirectional regulation, i.e. augmented PV expression lowering the mitochondria volume and conversely, increased mitochondria biogenesis leading to decreased PV levels is operational in many systems including kidney epithelial cells^24,25^, myotubes^32^, fast-twitch skeletal muscles^22,42^ and in the nervous system, in striatal neurons^43^ and Purkinje cells^23^. Changes in PV expression also occur in a neuron activity-regulated manner^44^, or resulting from impairment of the PV-expressing neuron population e.g. seen as a reduction in PV^+^ neurons possibly resulting from PV downregulation in schizophrenia^36,45^. Moreover, following brain injury, PV upregulation occurs in ependymal cells and reactive astrocytes in the lateral ventricle wall^19^ and in aging-related lateral ventricle wall stenosis^20^. In glial cells PV upregulation is discussed as a means to protect these cells from injury-induced mechanisms including hypoxic-ischemic insults, similarly as elevated PV levels were suggested to confer resistance against injury-induced cell death and excitotoxicity in motoneurons^46^. CG4 cells were used due to their resemblance to oligodendrocyte progenitor cells (OPC), when cultured in non-differentiating medium and the possibility to differentiate the cells into oligodendrocyte-like cells when maintained in differentiating medium^47^. The differentiation process is induced by a number of previously identified intrinsic and/or extrinsic factors^48–52^. Initial results revealed parental CG4 cells to be negative for PV expression, although low levels of *Pvalb* mRNA were present in both non-differentiated and differentiated CG4 cells. The little amounts of *Pvalb* transcripts were efficiently downregulated by the LV sh*Pvalb* approach, however in none of the experiments substantial differences between C-CG4 and shPV-CG4 were observed, indicating that neither *Pvalb* transcripts nor the possibly minute PV expression levels were of functional relevance. This is in line with previous studies reporting no PV expression either in glial cells or in progenitor cells^53^. Nonetheless, the low amount of *Pvalb* mRNA in C-CG4 cells might still hint towards a role for PV and/or *Pvalb* mRNA in oligodendrocyte progenitor/mature cell physiology under certain conditions that were not manifest in the experiments carried out in this study. While UV-induced ROS generation for a period of 10 min was most probably too short to substantially elevate *Pvalb* transcripts and even more so PV protein levels in C-and GFP-CG4 cells, the hypoxic conditions lasting for 72 h might be sufficiently long to affect *Pvalb* transcript and or PV protein levels. However, no changes in either *Pvalb* mRNA or PV protein levels were observed in hypoxia-challenged C-, GFP- and PV-CG4 cells. As a negative control, i.e. to exclude putative alterations caused lentivirus (LV) infection, CG4 cells transduced with a LV leading to GFP expression were used. In all experiments, no differences between C-CG4 and GFP-CG4 cells were observed precluding that effects observed in PV-CG4 cells were the result of the LV approach. To investigate the effects of PV in CG4 cells, cells overexpressing PV (PV-CG4) were always compared to both, C-CG4 (parental) and GFP-CG4 cells.

EF-hand proteins similar to PV such as the related Ca^2+^ buffer protein calretinin (CR) affect basic cellular properties: overexpression or absence of CR in mouse primary mesothelial cells inversely affects proliferation and cell migration^54^ and CR down-regulation in mesothelioma cells decreases cell viability^26^. Since measurements of other parameters (e.g. “wound closure” in the scratch assay) are affected by the proliferation rate, we ascertained that proliferation (and mobility) of CG4 cells were hardly affected by PV overexpression. More precisely, *in vitro* growth curves were not different in PV-CG4 and C-CG4 cells and there was no indication of altered cell viability, while the closing of a scratch occurred slightly slower in PV-overexpressing cells, possibly linked to the fact that PV-CG4 cells have shorter processes and thus cover a smaller area (see below). Thus, alterations brought about by particular Ca^2+^-binding proteins appear to be protein-specific and effects caused by a particular protein cannot be completely mimicked by any other Ca^2+^-binding protein^25,39,40^, also seen in mesothelioma cells^55^.

### GC4 cell morphology changes mediated by PV expression

With respect to mitochondria results obtained in cell bodies of CG4 cells, they were essentially identical as was observed previously in epithelial MDCK cells^24,25^. The presence of PV in PV-CG4 cells resulted in smaller cells with smaller nuclei and smaller soma cytoplasmic volumes, yet with unaltered ratios of volume_nuclei_/volume_cyto_. More importantly, the mitochondria volume in the cell body was decreased and moreover to a larger extent than the other parameters, i.e. the ratio volume_mito_/volume_cyto_ was still lower in PV-CG4 cells than in the controls (C-CG4 and GFP-CG4). This held true for both, OPC- and OLG-like CG4 cells. In agreement, levels of mitochondrial transcripts (*COX*1, *COX4/1*) and protein (COX I) were decreased in PV-overexpressing cells. This is most probably due to reduced mitochondria biogenesis, since transcript levels of genes implicated in mitochondria biogenesis, NRF1 (*NRF1*) binding to PGC-1α and the NRF1/2 target gene *Tfam*, implicated in transcription and replication of mtDNA were down-regulated in PV-CG4 cells, as were transcript levels of the master regulator of mitochondria biogenesis PGC-1α (*PPARGC1A*).

### Branching of CG4 cell processes affected by PV - a general effect linked to PV expression?

While morphological changes associated with PV-mediated mitochondria downregulation were previously observed in cell bodies of epithelial MDCK cells^25^, the effects of PV on process formation were hitherto unknown. In undifferentiated PV-CG4 OPC-like cells characterized by a bipolar morphology, cellular processes were clearly shorter than in the parental C-CG4 cells. Correspondingly in the differentiated multipolar PV-CG4 OLG-like cells, the number of intersections, as well as 2^nd^ and 3^rd^ order branches –evidenced by Sholl analysis– were significantly lower than in the same cells without PV expression. For process’ growth and branching, both Ca^2+^ signaling and moreover mitochondria play an important role as was shown for neuronal cell differentiation^28,56^. Both lengthening of processes and branching require high ATP production provided by the respiratory function of mitochondria. In chick embryonic sensory neurons, a stable filopodium within an axonal process, stalled mitochondria at the same site and the external signal NGF are sufficient to result in axon branching; this process is inhibited by blocking mitochondrial respiration or ablation of mitochondria^57,58^, reviewed in^59^. The same mechanism was reported in rat primary dentate granule cell cultures additionally highlighting the importance of AMPK in the axonal transport of mitochondria^60^. In line, mitochondrial density in processes of undifferentiated, as well as in differentiated CG4 cells was decreased in PV-overexpressing cells, providing an explication for the shorter processes. Of note, in several studies the importance of extracellular signals such as NGF, BDNF and chondroitin sulfate proteoglycans for axon branching and also for branching of oligodendrocyte processes was reported (reviewed in^61,62^). However, BDNF is mostly involved in the late stages of myelin repair, i.e. the postmitotic remyelination process. Since both undifferentiated and differentiated CG4 cells used in this study are proliferating cells, the lack of endogenous transcripts coding for BDNF-signaling molecules (BDNF, NGF, TrkB) in CG4 cells was not unexpected.

Assuming that ECM components and extracellular signaling molecules in the medium were not different in cultures from C-CG4 and PV-CG4 cells would indicate that the observed changes in process’ branching in CG4 cells is essentially linked to PV and altered mitochondria density and as such a cell-intrinsic process. Interestingly, changes in branching modulated by PV (and associated changes in mitochondria) appear not to be restricted to oligodendrocytes, but might equally well operate in neurons. In brain sections from PV−/− striatal “PV-expressing” (Pvalb) interneurons the number of dendritic intersections in the range from 50–150 µm is increased, as well as the number of 2^nd^–5^th^ order branches^18^. Whether mitochondrial volumes in these Pvalb neurons (soma and dendrites) that are lacking PV, i.e. from PV−/− mice, are increased, remains to be shown. Yet, the mitochondrial volume in Purkinje cell somata of PV−/− mice is increased, demonstrating that the PV/mitochondria antagonism is also functional in neurons^23^. In support, the mitochondrial volume is decreased in neurons of Thy-PV mice ectopically expressing PV in all neurons^43^ similar to the situation prevailing in PV-CG4 cells.

### PV-mediated reduction in mitochondria decreases acute ROS production, but has no effect on hypoxia-induced CG4 cell impairment

Changes in mitochondrial volume are associated with a wide range of important biological functions such as modification of oxidative capacity, apoptosis and other forms of cell death^63,64^. Mitochondria are the major source of intracellular reactive oxygen species (ROS) and excessive mitochondria-generated ROS leads to peroxidation of lipids, DNA damage and oxidative impairment of macromolecules implicated in cell survival, subsequently leading to activation of apoptotic pathways^65^. While ROS generation in CG4 cells with or without PV expression under basal conditions was similar despite clear differences in mitochondrial volume, ROS generation induced by short, but intensive UV light exposure was significantly higher in C-CG4 cells with a larger fractional mitochondrial volume than in PV-CG4 cells. This indicates that the lower mitochondrial density resulting from PV expression decreased UV-induced ROS production and might thus be considered as a protective mechanism in “oxidative stress conditions” such as hypoxic-ischemic conditions. Of note differences in ROS production –evidenced by measurements of DHE red fluorescence– were only significant in the branched differentiated OLG-like CG4 cells indicating that specifically cells with a more mature oligodendrocyte-like phenotype were generating more UV-induced ROS and might thus be more vulnerable to ROS cytotoxicity. The oligodendrocyte lineage of glial cells is more sensitive to an altered redox state than other glial types, possibly due to their high lipid and iron content, decreased levels of antioxidant enzymes or limited substrates^66^. With respect to oligodendrocyte differentiation status, immature OPCs are more vulnerable to hypoxic-ischemic insults *in vitro* than are more mature oligodendrocytes (reviewed in^11^). Although cell viability was decreased after 72 h of hypoxia in CG4 cells, the decrease in viability was similar in cells in the OPC-like and OLG-like state and moreover was not different in cells with or without PV expression. Although also hypoxia-mediated injury is linked to disruption/impairment of mitochondrial function and Ca^2+^ signaling (reviewed in^11^), in our experimental conditions no differences with respect to cell viability were observed, despite significant differences in mitochondrial volume. Since hypoxia is also associated with (glutamate-mediated) increase in the intracellular Ca^2+^ concentration leading to the activation of Ca^2+^ -dependent proteases, lipases and moreover that PV protects motoneurons from excitotoxicity^46,67^, it is hypothesized that either increased mitochondria (C-CG4 cells) or increased PV (PV-CG4 cells) might exert a protective effect against hypoxia of similar magnitude. In support, we have found no evidence for either hypoxia-induced PV down-regulation in PV-CG4 cells nor the induction of PV expression in C-and GFP-CG4 cells, both of which would have been an explication for the absence of a presumably protective effect observed in hypoxia-treated PV-CG4 cells. A more detailed analysis of this aspect may be necessary (milder hypoxic conditions, shorter duration) before firmly claiming that PV levels are not linked to hypoxia-induced impairment of cell viability. As the excessive release of ROS seem to play an important role in the pathogenesis of multiple sclerosis and contribute to myelin damage and axon degeneration^9,68^, PV upregulation in mature oligodendrocytes might be beneficial in counteracting these processes. Also the question of low PV levels and increased ROS production warrants further studies. Augmented ROS production impairs the function of PV-expressing neurons in mouse models of schizophrenia^36,45^ and moreover decreases in PV levels are observed in the brains of schizophrenia patients^69^ and schizophrenia mouse models^45^. Thus, PV-mediated changes in mitochondrial volumes in Pvalb neurons provide an interesting link to this study.

## Supplementary information

Supplementary Material

## Data Availability

Authors make materials, data and associated protocols available to readers without undue qualifications in material transfer agreements. The datasets generated and analyzed during the current study are available in the ZENODO repository, (10.5281/zenodo.3335842), https://zenodo.org/deposit/3335842
