# Author Correction: Parvalbumin expression in oligodendrocyte-like CG4 cells causes a reduction in mitochondrial volume, attenuation in reactive oxygen species production and a decrease in cell processes’ length and branching

**DOI:** 10.1038/s41598-020-69593-9

**Published:** 2020-07-22

**Authors:** Lucia Lichvarova, Walter Blum, Beat Schwaller, Viktoria Szabolcsi

**Affiliations:** Anatomy, Section of Medicine, University of Fribourg, Route Albert-Gockel 1, CH-1700 Fribourg, Switzerland

Correction to: *Scientific Reports* https://doi.org/10.1038/s41598-019-47112-9, published online 22 July 2019

This Article contains errors.

The original source of Fig. 1A, B, G, and H could not be identified. A revised version of Figure 1 appears below, with the corresponding movies, which are available in the ZENODO repository https://doi.org/10.5281/zenodo.3907289^1^, referenced in the figure legend.

**Figure 1:**
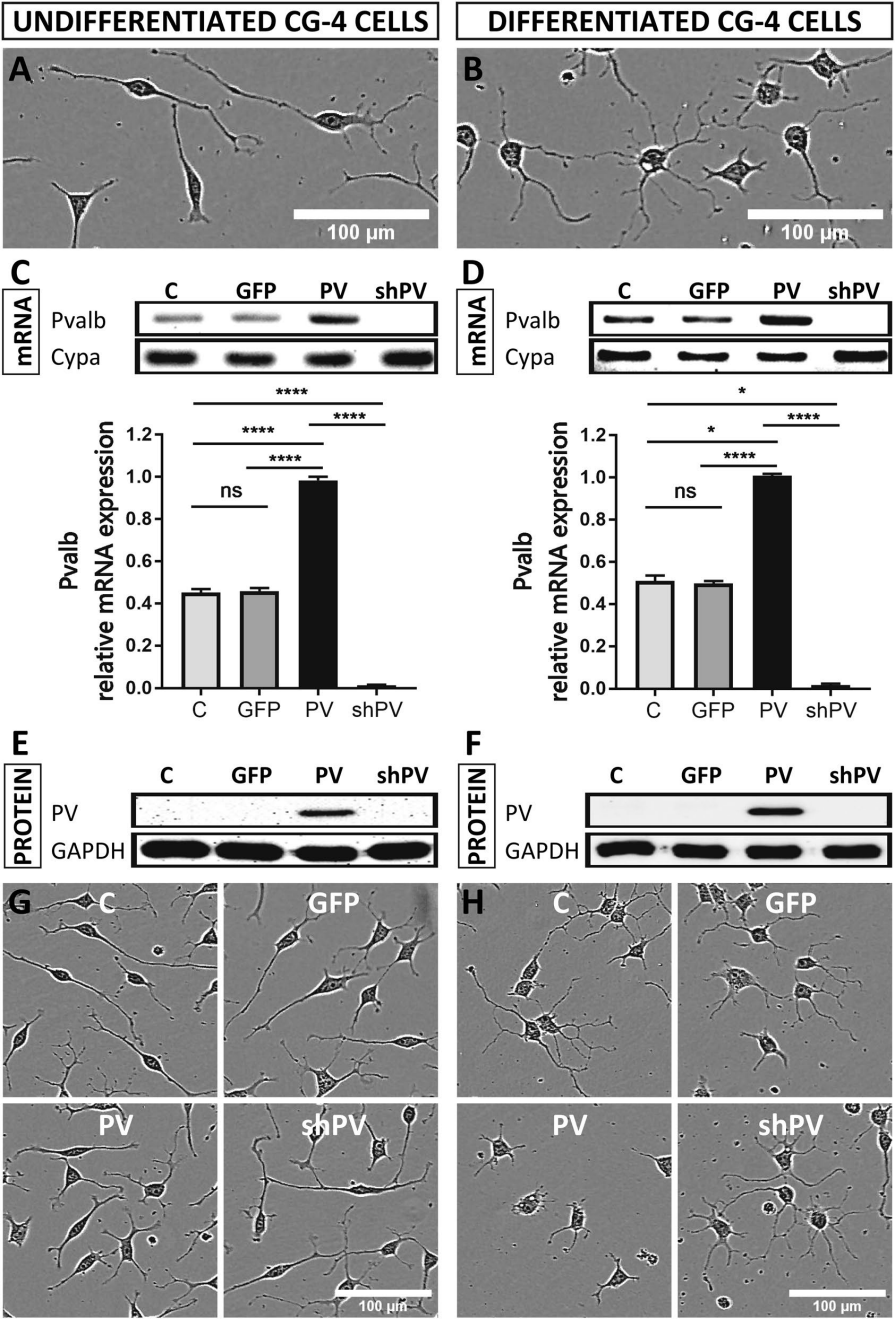
CG4 cells display an OPCs-like morphology in the undifferentiated state (**A**) and oligodendrocyte-like phenotype in the differentiated state (**B**). PV (*Pvalb*) transcript levels were determined by semi-quantitative PCR after 30 cycles (upper) and by RT-qPCR (lower), where *Cypa* was used as reference gene (**C**,**D**). PV expression levels were determined by Western blot analyses, where GAPDH signals were used as loading control (**E**,**F**). The morphological features of LV-infected CG4 cells (GFP, PV, sh*PV*) appeared similar to the control (C) in both undifferentiated OPC-like and differentiated OLG-like states (**G**,**H**). Images A, B, G and H were acquired with the IncuCyte system. The same raw data (movie) files were also used to select representative cells shown in Figs. 6A, B and 7A, B. All movie files are available in the ZENODO repository https://doi.org/10.5281/zenodo.3907289^1^. The individual frames from which the representative cells were taken are marked and annotated in the “Supporting Information” (.pdf) file available in the same repository.

Figure 3A (control start point) was a duplication of Figure 3B (control start point). The correct Figure 3 appears below as Figure 2, and a link to the original data has been added in the figure legend.

**Figure 2:**
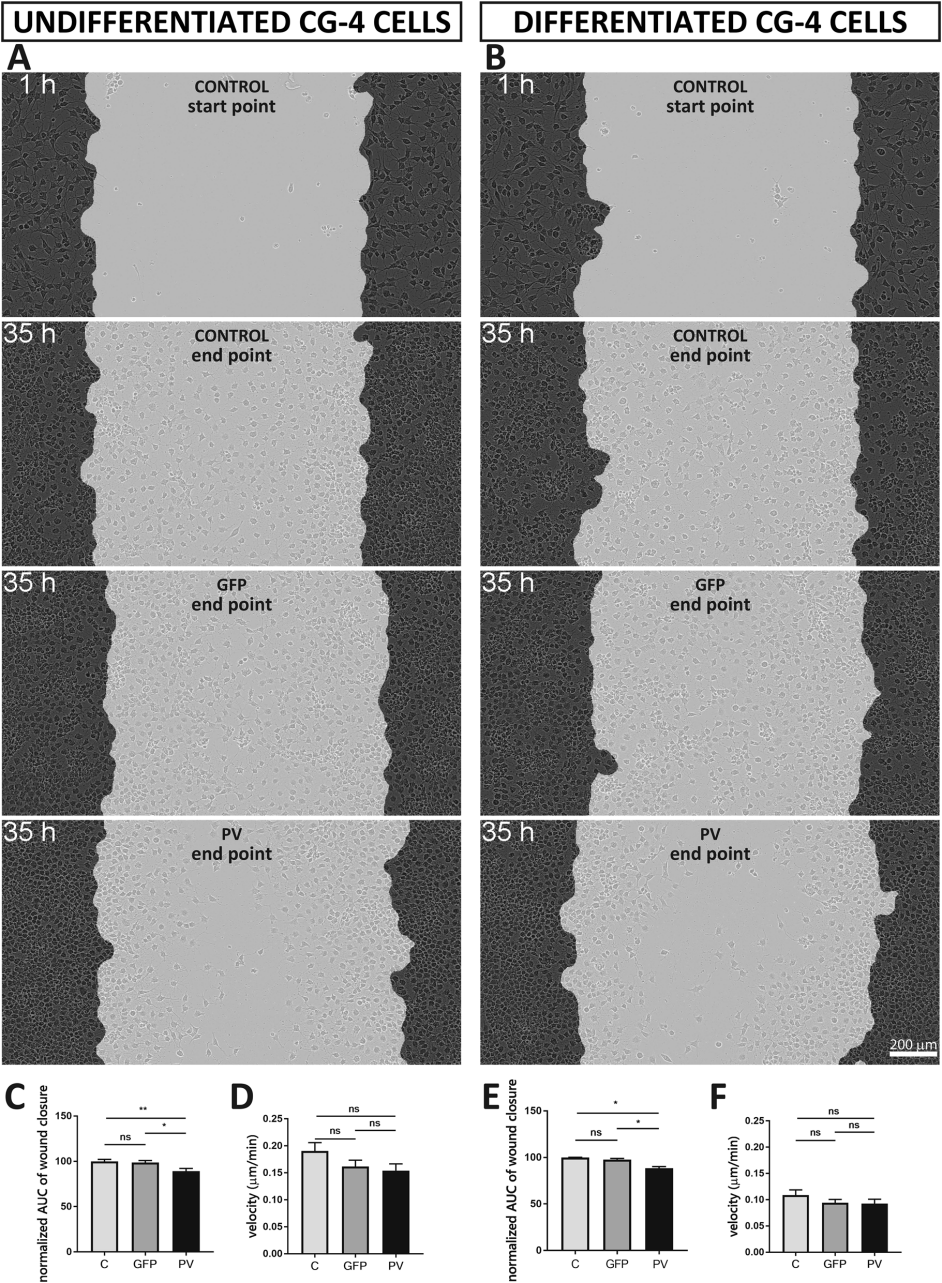
PV expression affected “wound closure” capacity as assessed by a scratch wound assay using undifferentiated C-, GFP- and PV-CG4 cells (**A**) and the same differentiated CG4 cell lines (**B**). All movie files from which the images were taken are available in the ZENODO repository https://doi.org/10.5281/zenodo.3754905^2^. The wound closure capacity was evaluated by calculating the AUC of the relative wound density plotted over time, which was normalized to the control (C-CG4 cells) and averaged per condition. In the undifferentiated OPC state PV-CG4 cells displayed slightly decreased AUC values (relative wound density over time) when compared to C-CG4 cells (**C**), suggesting a reduced wound closure capacity in PV-overexpressing CG4 cells in the undifferentiated state. Similar differences were also present in CG4 cells in the differentiated state (**E**). Single-cell tracking from video sequences revealed no significant differences in the cell velocity (µm/min) of CG4 cells, in both the undifferentiated OPC-like (**D**) and differentiated OLG-like states (**F**). Error bars represent SEM (ns: not significant; *p* value > 0.05, *p < 0.05, **p < 0.001).

The cell depicted as a representative PV-CG4 cell, shown in Fig. 6A, was selected erroneously from a movie frame of GFP-CG4 cells, shown in Fig. 7A. A correct version of Figure 6 appears below as Figure 3, and a link to the original data has been added to the figure legend.

**Figure 3:**
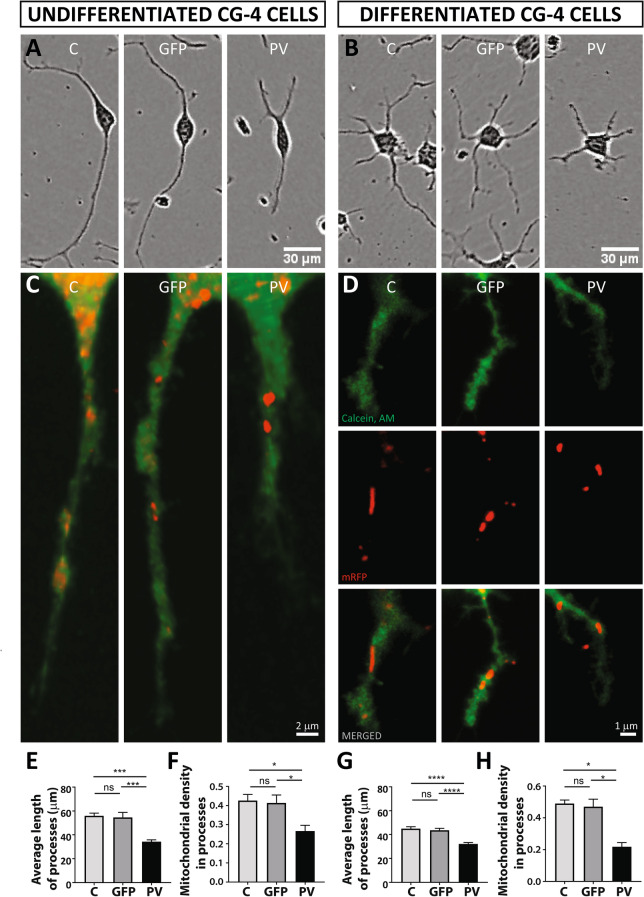
PV overexpression affects mitochondrial density and position of mitochondria in distal cellular processes. Representative CG4 cells are depicted from IncuCyte images (**A**,**B**) The raw data (movies) are available in the ZENODO repository https://doi.org/10.5281/zenodo.3907289^1^. The individual frames from which the representative cells were taken are marked and annotated in the “Supporting Information” (.pdf) file available in the same repository. Mitochondria were visualized by mitochondria-targeted red fluorescent protein (red) and cellular processes were visualized by Calcein AM (green). Representative images are shown for C-, GFP- and PV-CG4 cells (**C**,**D**). In **D**) separate images for cytoplasm (green, upper), mitochondria (red, middle) and merged images (lower) are shown. In addition, average length of processes (**E**,**G**) and mitochondrial density in the processes (**F**,**H**) was analyzed. Cellular process length of CG4 cells was evaluated by automatically acquired real-time images by the IncuCyte Imaging system. Single cells were analyzed and the length of cellular processes was measured. Average process’ length was evaluated by measuring total length of all processes per cell and dividing this number by the number of processes in the same cell. Error bars represent SEM, *p < 0.05, **p < 0.01, ***p < 0.001, ****p < 0.0001.

In Figure 7, the specified time points (6, 12 and 24 h) of representative IncuCyte images for undifferentiated (**A**) and differentiated (**B**) CG4 cells were incorrect. A corrected version of Figure 7 appears below as Figure 4, with the source of the data provided in the figure legend.

**Figure 4:**
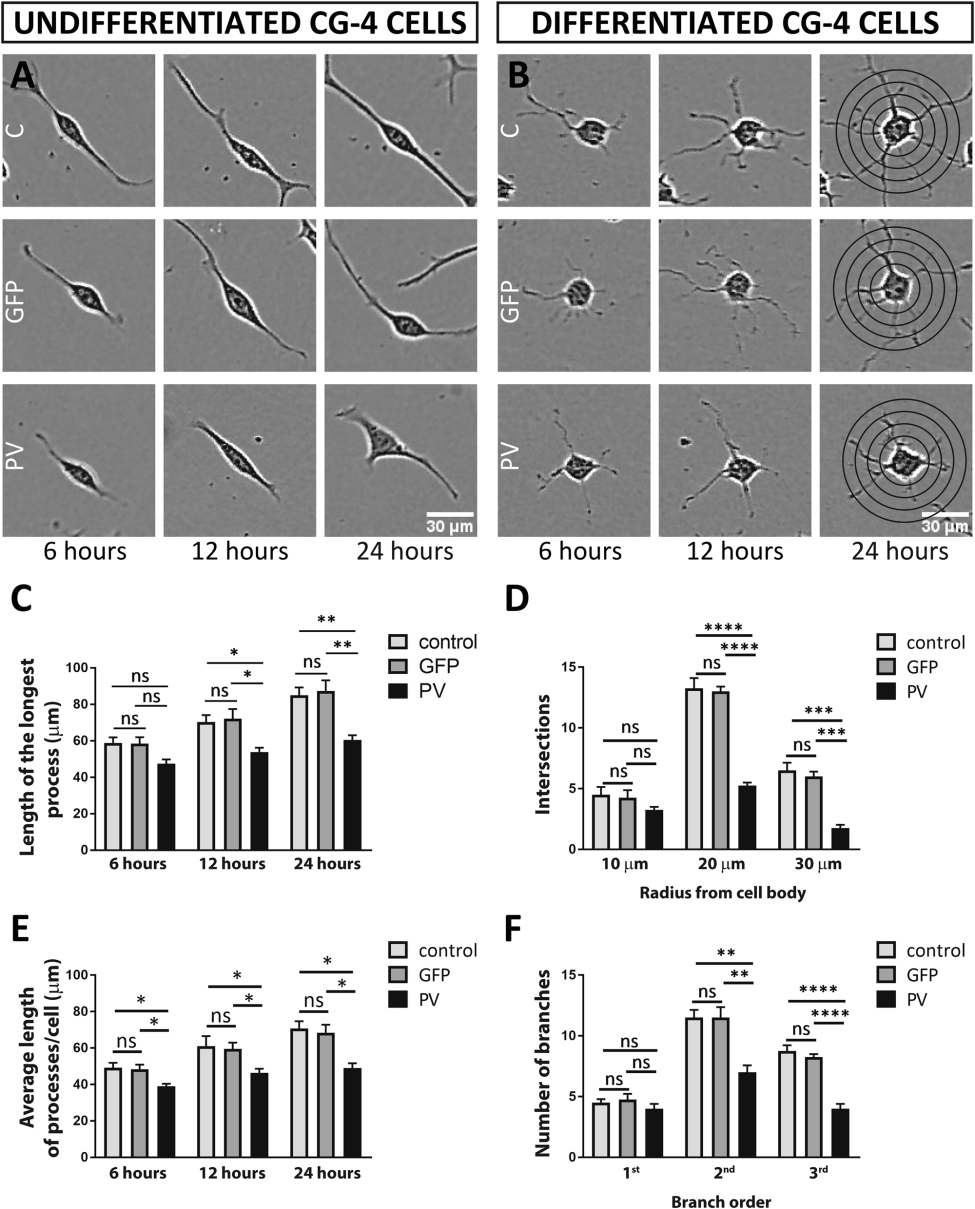
Length of processes in undifferentiated CG4 cells and Sholl analyses of process’ branching in differentiated CG4 cells. Representative undifferentiated (**A**) and differentiated (**B**) CG4 cells are depicted from IncuCyte images obtained at 6, 12 and 24 h after seeding. The raw data (movies) are available in the ZENODO repository https://doi.org/10.5281/zenodo.3907289^1^. The individual frames from which the representative cells were taken are marked and annotated in the “Supporting Information” (.pdf) file available in the same repository. For the length of the longest process (**C**) and average length of processes/cell (**E**) all time points (6, 12, 24 h) are shown. Sholl analyses of process’ branching in differentiated CG4 cells is shown for 24 h after seeding (**D, F**). The number of intersections (**D**) was analyzed at a 10-, 20- and 30-µm radius from the cell body. Number of branches (**F**) was analyzed for first, second and third branch order (n.s. = not significant, *p < 0.05, **p < 0.01, ***p < 0.001, ****p < 0.0001).

The text in the Data Availability statement,

“The datasets generated and analyzed during the current study are available in the ZENODO repository, (https://doi.org/10.5281/zenodo.3335842)”

should read:

“The datasets generated and analyzed during the current study are available in the ZENODO repositories https://doi.org/10.5281/zenodo.3907289 (representative movies for images shown in Figs. 1, 6 and 7), https://doi.org/10.5281/zenodo.3754905 (representative movies for images shown in Fig. 3), and https://doi.org/10.5281/zenodo.3335842 (all other data).”
