# Supplementary material for: Parvalbumin expression in oligodendrocyte-like CG4 cells causes a reduction in mitochondrial volume, attenuation in reactive oxygen species production and a decrease in cell processes’ length and branching
Source: Sci Rep. 2019 Jul 22;9:10603. doi: 10.1038/s41598-019-47112-9 (PMC6646370; doi:10.1038/s41598-019-47112-9)
Supplement: Supplementary file 1 — Supplementary Material [file 41598_2019_47112_MOESM1_ESM.pdf]

Parvalbumin expression in oligodendrocyte-like CG4 cells causes a reduction in mitochondrial volume, attenuation in reactive oxygen species production and a decrease in cell processes' length and branching

Lucia Lichvarova, Walter Blum, Beat Schwaller and Viktoria Szabolcsi

Supplementary Figures 1 -7

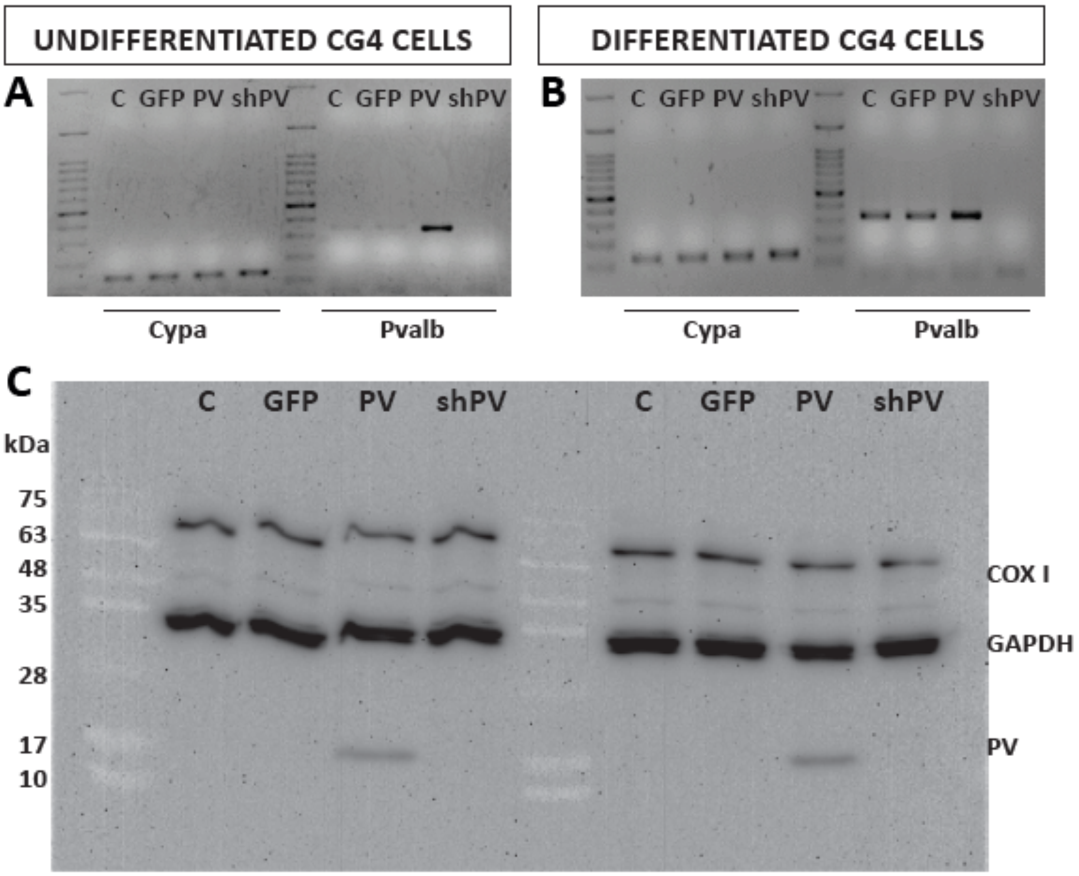

Supplemental Figure S1 (Supplementary information to Figure 1)
Unprocessed images of agarose gels with PCR samples from undifferentiated **(A)** and differentiated CG4 cells **(B)** taken by the FluorChem E System. Images correspond to Fig. 1C, D of the main text. Transcript levels of parvalbumin (*Pvalb* mRNA; product size 343 bp) were determined by semi-quantitative PCR after 30 cycles, whereas *Cypa* encoding cyclophylin A was used as reference gene (product size 126 bp). PV protein expression levels were determined by Western blot analyses. Unprocessed image, taken by the FluorChem E System **(C)**, shows the whole membrane containing samples from undifferentiated (left part of the blot) and differentiated CG4 cells (right part of the blot). This image corresponds to Fig. 1 E, F of the main text. Sample loading order was C, GFP, PV and shPV. GAPDH signals were used as loading control.

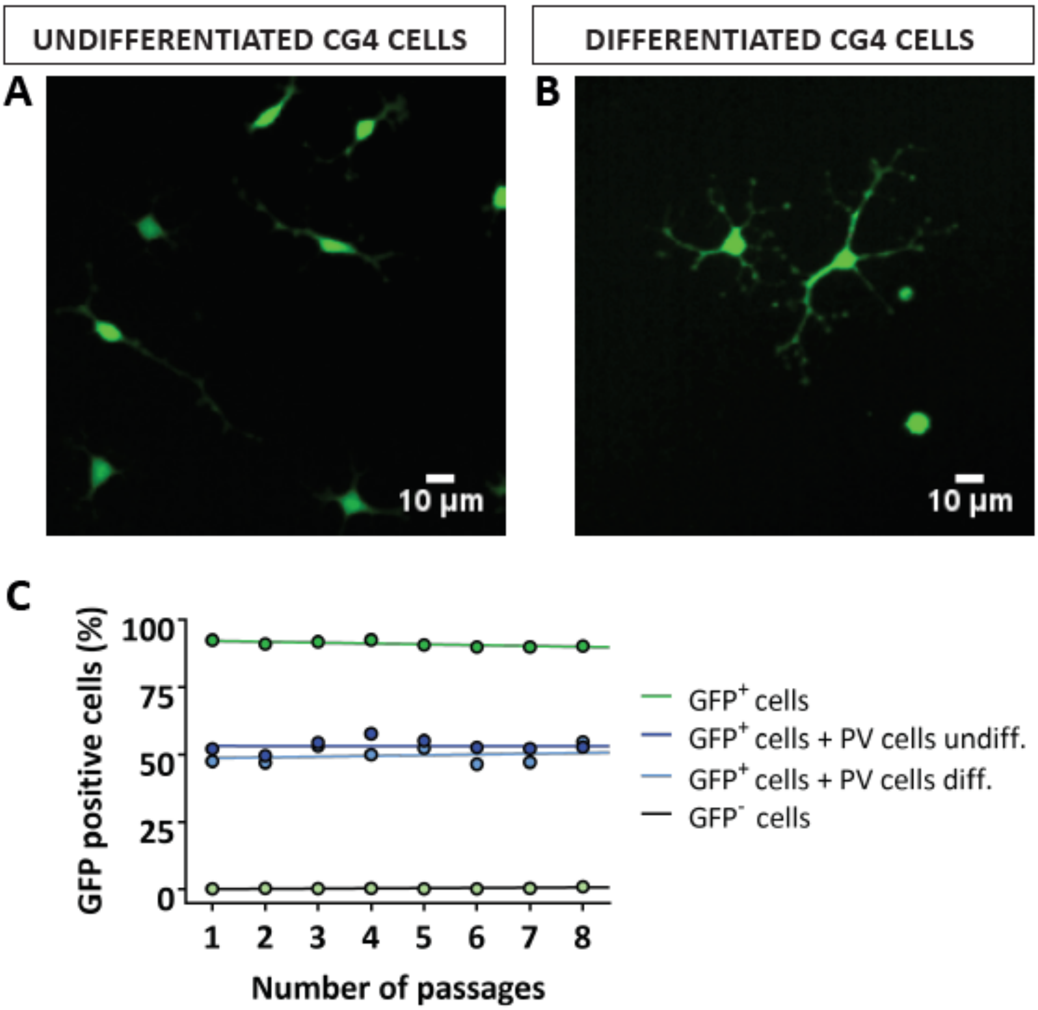

Supplemental Figure S2

Representative images of undifferentiated **(A)** and differentiated **(B)** CG4 cells transduced with LV producing the green fluorescent protein (GFP) are shown. A multiple passaging experiment was carried out with a mix of GFP-CG4 cells and PV-CG4 cells and a mix of GFP-CG4 and C-CG4 cells at a starting ratio of 50/50. The ratio of GFP+ cells at each passage by FACS analysis over several passages was analyzed **(C)**. No changes in the ratios were detected indicating that growth properties of the groups were unchanged over time.

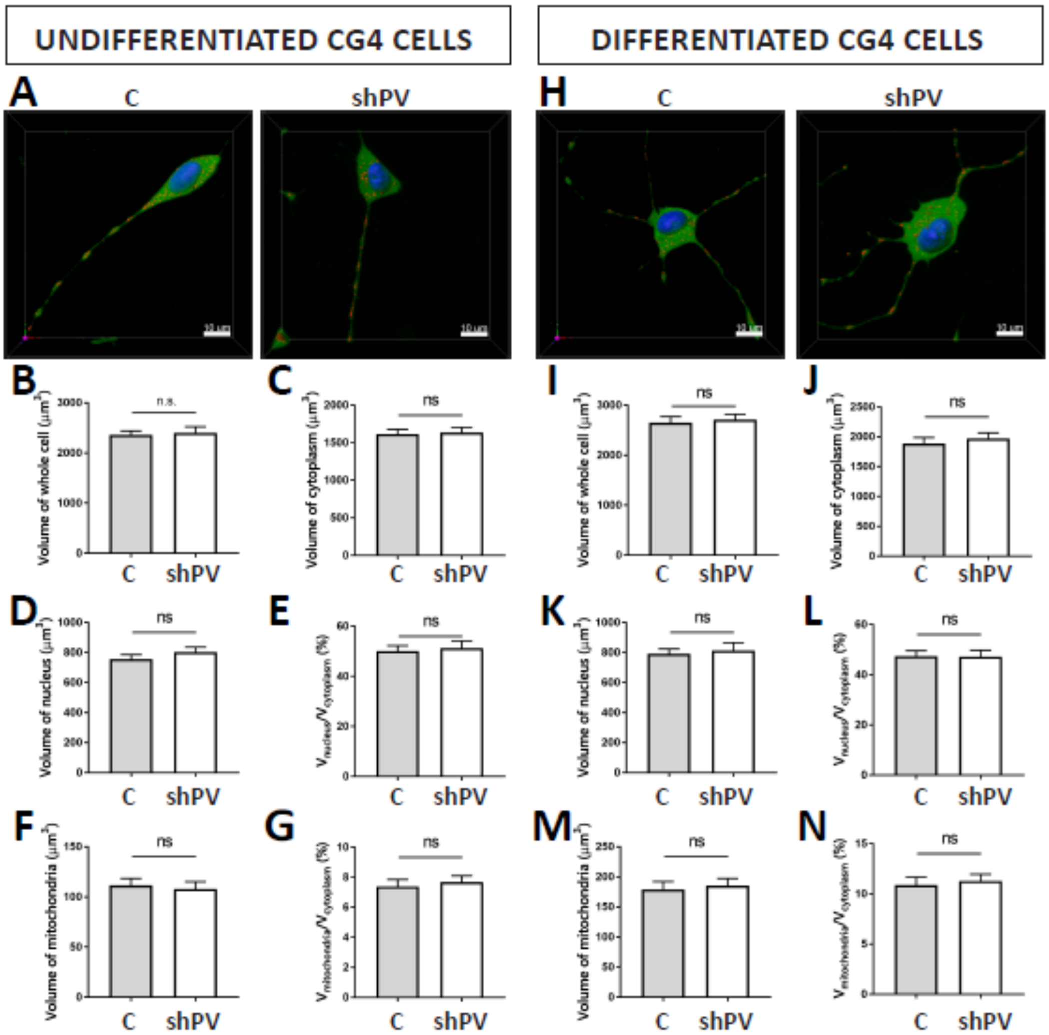

Supplemental Figure S3 (Supplementary information to Figure 4)

Representative CG4 cells (control and shPV) are shown in their undifferentiated **(A)** and differentiated **(H)** states. CG4 cells were loaded with MTR CMXRos (red), Hoechst (blue) and Calcein, AM (green) and z-stack images were imaged by confocal microscopy to determine volumes of interest. Quantification of soma cell volume **(B, I)**, soma cytoplasmic volume **(C, J)**, volume of nucleus **(D, K)**, ratio of nucleus/soma cytoplasm **(E, L)**, soma mitochondrial volume **(F, M)** and ratio of soma mitochondria/soma cytoplasm **(G, N)** was performed using the Imaris software.

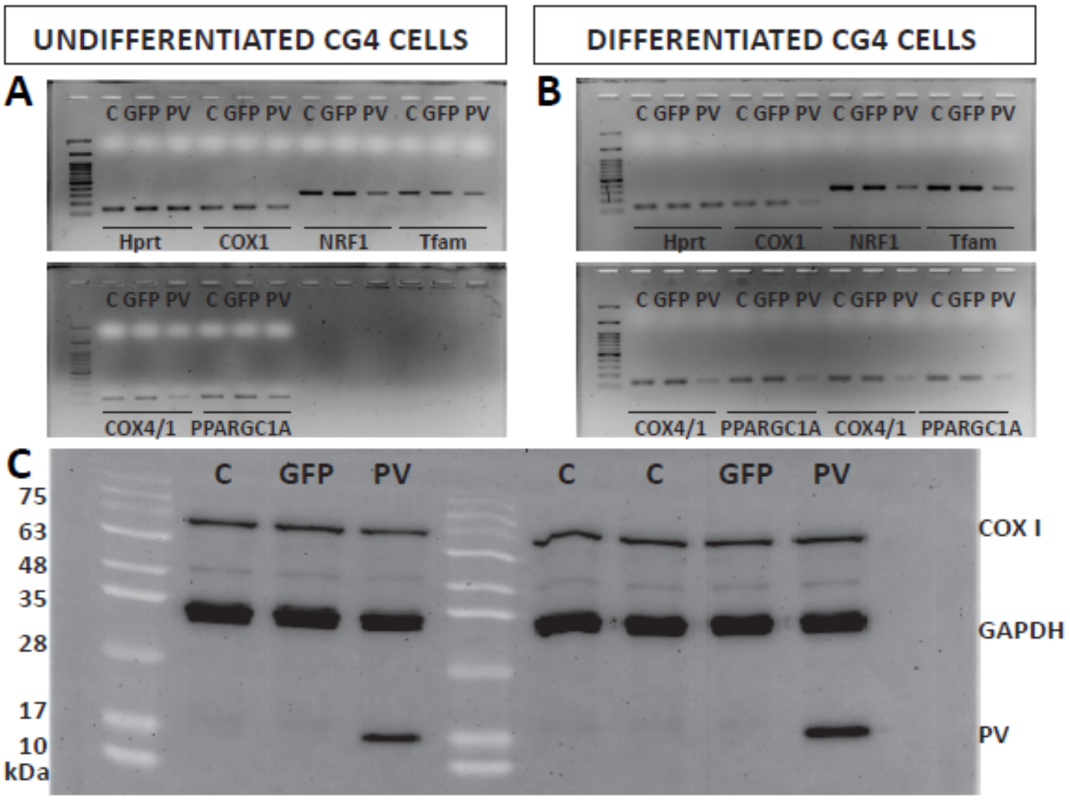

Supplemental Figure S4 (Supplementary information to Figure 5)

Unprocessed images of the entire agarose gels of undifferentiated **(A)** and differentiated **(B)** CG4 cells taken by the FluorChem E System. Images correspond to Fig. 5A, C, E-L of the main text. Transcript levels of *COX1*, *NRF1*, *Tfam*, *COX4/1* and *PPARGC1A* (names marked below bands) were determined by semi-quantitative PCR, where *Hprt* was used as reference gene. Samples were loaded in the order: C, GFP, PV; for all genes. Unprocessed image of a Western blot **(C)** taken by the FluorChem E System shows the entire nitrocellulose membrane loaded with samples from undifferentiated (left part of the blot) and differentiated CG4 cells (right part of the blot). This image corresponds to Fig. 5 B, D of the main text. Sample order: C, GFP, PV; for samples from undifferentiated CG4 cells and C, C, GFP, PV; for samples from differentiated CG4 cells. GAPDH signals were used as loading control. Single bands of the correct size are seen for COX I, GAPDH and PV.

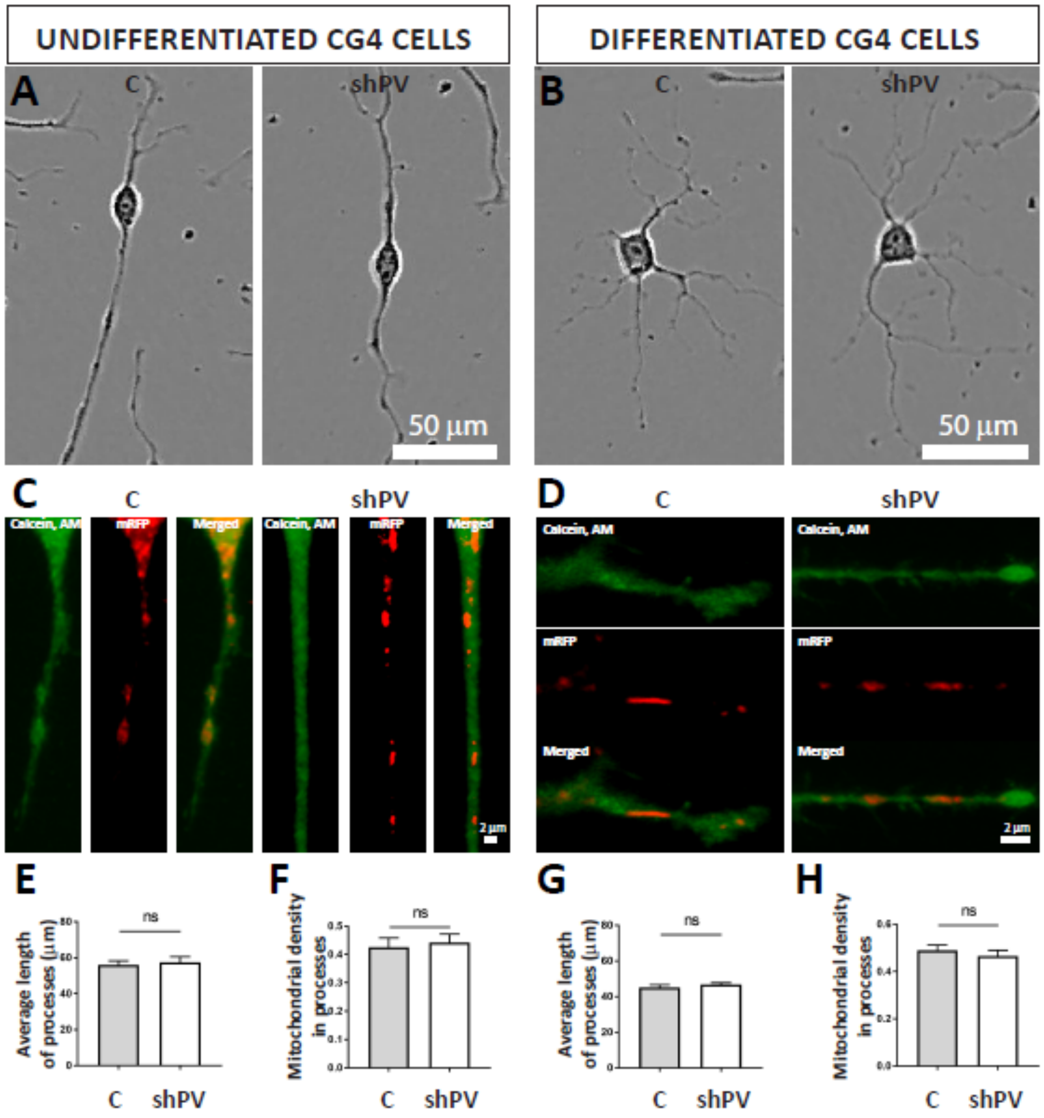

Supplemental Figure S5 (Supplementary information to Figure 6)

Representative CG4 cells (C- and shPV-) are depicted; taken from IncuCyte images **(A, B)**. CG4 cells were transfected with mitochondria-targeted red fluorescent protein (mRFP) in order to visualize mitochondria and distal cellular processes were visualized with Calcein-AM **(C, D)**. Analysis of average length of processes **(E, G)**, as well as analysis of mitochondrial density in CG4 cell processes **(F, H)**.

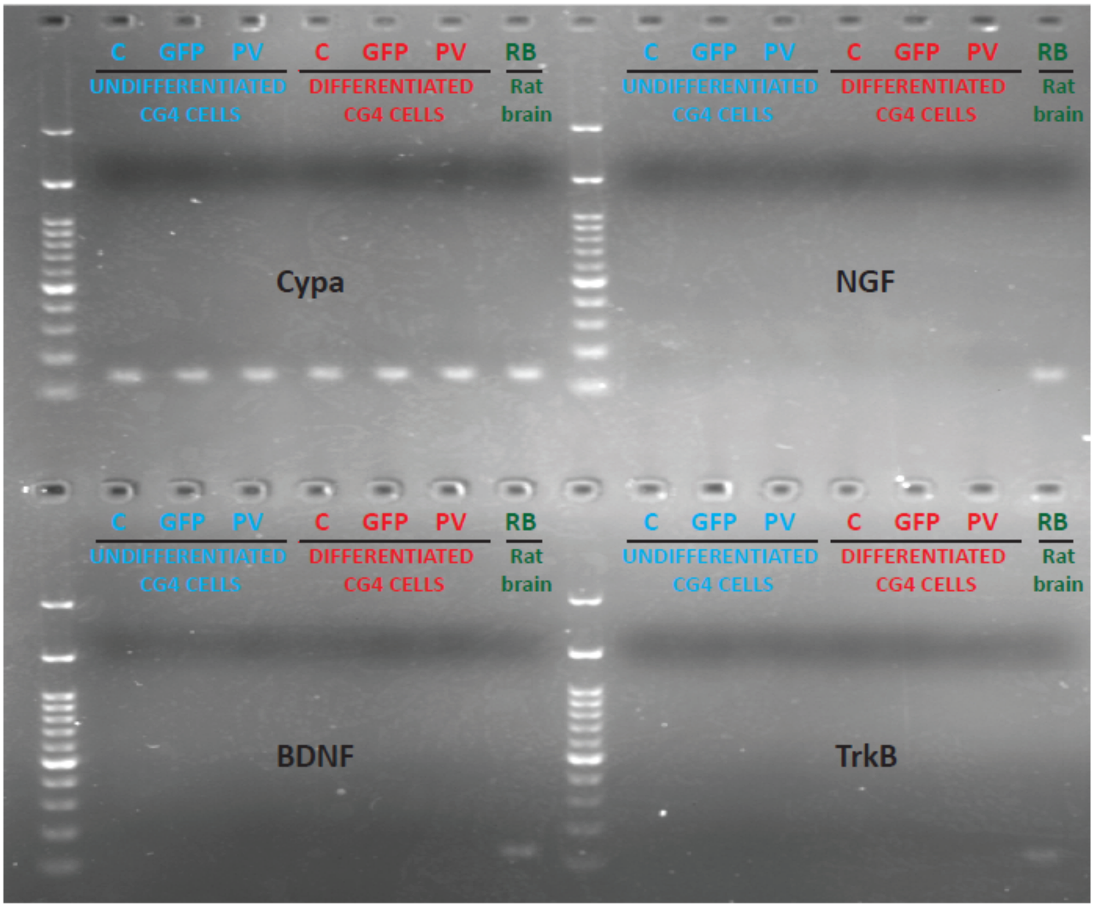

Supplemental Figure S6

Unprocessed images of agarose gels with PCR samples from undifferentiated and differentiated CG4 cells, as well as rat forebrain (serving as positive control) taken by the FluorChem E System. Transcript levels of BDNF (*Bdnf*), NGF (*Ngf*), TrkB (*Ntrk2*) (marked below bands) were determined by semi-quantitative PCR, where *Cypa* was used as reference gene. Samples were loaded in the order: undifferentiated CG4 cells C, GFP, PV; differentiated CG4 cells C, GFP, PV and rat forebrain; for all genes.

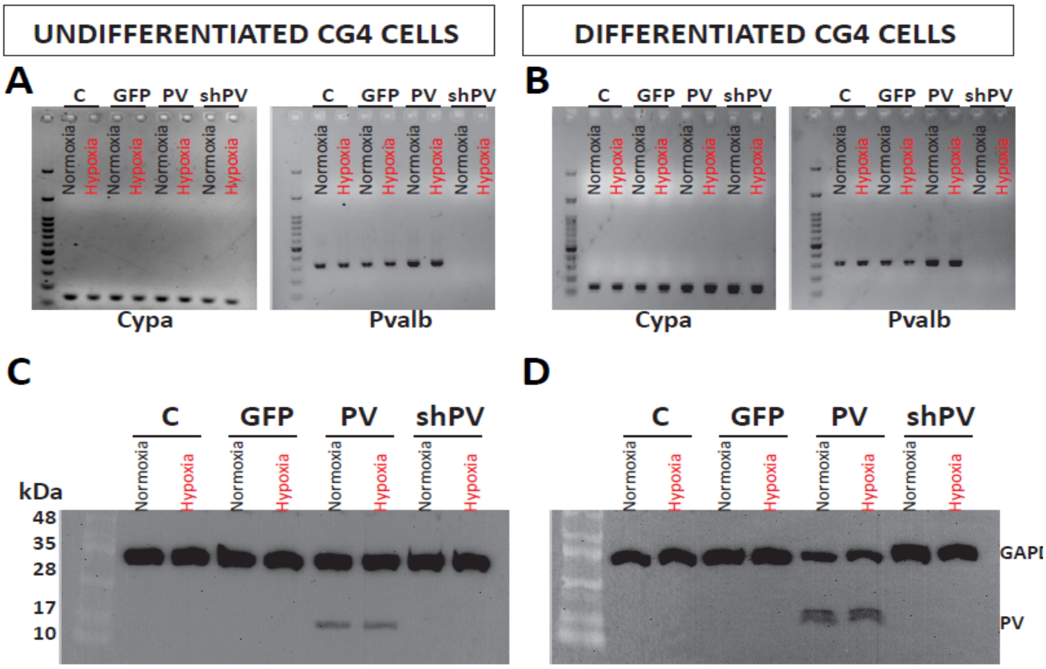

Supplemental Figure S7 (Supplementary information to Figure 8)
Unprocessed images of agarose gels with PCR samples from undifferentiated **(A)** and differentiated CG4 cells **(B)** taken by the FluorChem E System. Images correspond to Fig. 8G, H of the main text. Transcript levels in normoxic as well as hypoxic conditions were determined by semi-quantitative PCR after 30 cycles. *Cypa* encoding cyclophylin A was used as reference gene. PV protein expression levels were determined by Western blot analyses. Unprocessed images, taken by the FluorChem E System, show the whole membrane containing samples from undifferentiated **(C)** and differentiated **(D)** CG4 cells. GAPDH signals were used as loading control. This image corresponds to Fig. 8 I, J of the main text.
